# Overexpression of *OsTF1L,* a rice HD‐Zip transcription factor, promotes lignin biosynthesis and stomatal closure that improves drought tolerance

**DOI:** 10.1111/pbi.12951

**Published:** 2018-07-19

**Authors:** Seung Woon Bang, Dong‐Keun Lee, Harin Jung, Pil Joong Chung, Youn Shic Kim, Yang Do Choi, Joo‐Won Suh, Ju‐Kon Kim

**Affiliations:** ^1^ Graduate School of International Agricultural Technology and Crop Biotechnology Institute/GreenBio Science and Technology Seoul National University Pyeongchang Korea; ^2^ Center for Nutraceutical and Pharmaceutical Materials Division of Bioinformatics Myongji University Yongin, Gyeonggi Korea; ^3^ Department of Agricultural Biotechnology Seoul National University Seoul Korea; ^4^ Present address: NUS Synthetic Biology for Clinical and Technological Innovation Department of Biochemistry Yong Loo Lin School of Medicine National University of Singapore Singapore 117596 Singapore

**Keywords:** drought tolerance, *Oryza sativa*, *OsTF1L*, lignin biosynthesis, stomatal closure, HD‐Zip transcription factor

## Abstract

Drought stress seriously impacts on plant development and productivity. Improvement of drought tolerance without yield penalty is a great challenge in crop biotechnology. Here, we report that the rice (*Oryza sativa*) homeodomain‐leucine zipper transcription factor gene, *OsTF1L* (*Oryza sativa transcription factor 1‐like*), is a key regulator of drought tolerance mechanisms. Overexpression of the *OsTF1L* in rice significantly increased drought tolerance at the vegetative stages of growth and promoted both effective photosynthesis and a reduction in the water loss rate under drought conditions. Importantly, the *OsTF1L* overexpressing plants showed a higher drought tolerance at the reproductive stage of growth with a higher grain yield than nontransgenic controls under field‐drought conditions. Genomewide analysis of *OsTF1L* overexpression plants revealed up‐regulation of drought‐inducible, stomatal movement and lignin biosynthetic genes. Overexpression of *OsTF1L* promoted accumulation of lignin in shoots, whereas the RNAi lines showed opposite patterns of lignin accumulation. *OsTF1L* is mainly expressed in outer cell layers including the epidermis, and the vasculature of the shoots, which coincides with areas of lignification. In addition, *OsTF1L* overexpression enhances stomatal closure under drought conditions resulted in drought tolerance. More importantly, OsTF1L directly bound to the promoters of lignin biosynthesis and drought‐related genes involving *poxN/PRX38*,* Nodulin protein*, *DHHC4*, *CASPL5B1* and *AAA‐type ATPase*. Collectively, our results provide a new insight into the role of *OsTF1L* in enhancing drought tolerance through lignin biosynthesis and stomatal closure in rice.

## Introduction

Drought is an abiotic stress that induces a range of molecular, biochemical and physiological responses in plants, which in turn can result in a reduction in crop productivity (Pandey and Shukla, 2015). Consequently, there is considerable interest in developing drought‐tolerant crops that do not exhibit a yield penalty under normal growth conditions. Many studies have identified transcription factors (TFs) to be involved in the drought tolerance mechanisms. Modifying their activity may provide a means to increase the capacity of a plant to avoid drought stress. For example, a number of TFs from rice (*Oryza sativa*) have been shown to enhance drought tolerance using an overexpression strategy (Hu *et al*., 2006; Jeong *et al*., 2010, 2013; Lee *et al*., 2015, 2016, 2017; Oh *et al*., 2009; Park *et al*., 2015; Redillas *et al*., 2012).

All eukaryotic organisms have homeodomain (HD) type TFs that are characterized by 60 conserved amino acids, and plant‐specific homeodomain‐leucine zipper (HD‐Zip) TFs contain both a homeodomain and a leucine zipper domain (Ariel *et al*., 2007). These TFs play important roles in plant growth and development, as well as in stress responses. Among them, HD‐Zip class I TFs were well studied in stress tolerance mechanisms, such that overexpression of *HaHB4*,* HaHB1*,* AtHB13* and *Zmhdz10* in *Arabidopsis* enhances tolerance to drought and high salinity (Cabello and Chan, 2012; Cabello *et al*., 2007; Dezar *et al*., 2005; Manavella *et al*., 2006; Zhao *et al*., 2014). Furthermore, a member of HD‐Zip class IV, *Arabidopsis EDT1/HDG11* was identified as a key player to improve drought tolerance. Overexpression of *AtEDT1/HDG11* enhances tolerance to drought stress in rice, *Arabidopsis*, tobacco, cotton, pepper and Chinese kale via reduction in stomatal density, acceleration of stomatal closure and improvement of root system (Yu *et al*., 2008, 2013, 2016; Zhu *et al*., 2015; Zhu *et al*., 2016). However, the underlying molecular mechanisms of their drought tolerance are not well understood.

The rice HD‐Zip class IV consists of 11 members, which are named as *Rice outermost cell‐specific* (*Roc*) (Javelle *et al*., 2011) and *O. sativa transcription factor 1* (*OsTF1*) (Yang *et al*., 2002). Ito *et al*. (2002, 2003) demonstrated the epidermis‐predominant expression of five *Roc* genes, *Roc1*,* 2*,* 3*,* 4* and *5*, suggesting a potential role in epidermal differentiation. *OsTF1*, another member of the HD‐Zip class IV, exhibited a similar expression pattern during early embryogenesis (Yang *et al*., 2002). Of the 11 class IV genes, only *Roc 4* and *5* have been functionally characterized so far to be associated with flowering time (Wei *et al*., 2016), and with leaf rolling (Zou *et al*., 2011), respectively. Thus, it remains elusive to understand roles of the HD‐Zip class IV in development and abiotic stress responses of rice.

Many kinds of literature suggest that abiotic stress tolerance is connected to plant lignification. In *A. thaliana*, overexpression of *PaSOD* from *Potentilla atrosanguinea* and/or *RaAPX* from *Rheum austral* induces high lignification in inflorescence stems, conferring tolerance to salt and cold stress through up‐regulation of the lignin biosynthetic genes, *PAL1* and *PRXR9GE* (Gill *et al*., 2010; Shafi *et al*., 2014). In maize (*Zea mays*), drought‐tolerant inbred lines with high expression levels of lignin biosynthetic genes such as *cinnamyl alcohol dehydrogenase* (*CAD*), *cytochrome protein 96A8* (*COMT*) or *S‐adenosyl‐L‐methionine synthase* (*SAMS*; Hu *et al*., 2009) show highly lignified shoots, suggesting a correlation between drought tolerance and shoot lignification. Overexpression of the *IbLEA14* gene increases the lignin content in transgenic sweet potato (*Ipomoea batatas*) calli and enhances tolerance to drought and salt stress due to an up‐regulation of lignin biosynthetic genes (Park *et al*., 2011). In wild watermelon (*Citrullus lanatus*), which is notably drought‐tolerant, increase in expression levels of lignin biosynthetic genes has been observed, which may represent a survival strategy during progressive drought stress (Yoshimura *et al*., 2008). Embolism resistance is an indicator of plant response to drought and adaptive strategies under drought conditions (Lens *et al*., 2013). Greater lignin content in the xylem improves resistance to drought‐induced embolism (Pereira *et al*., 2018), while mutants with reduced lignin content showed increased embolism vulnerability (Awad *et al*., 2012; Coleman *et al*., 2008; Voelker *et al*., 2011). Thus, it appears that high levels of lignification in plants can lead to increased drought tolerance, although the associated regulatory networks have not been well elucidated.

Transpirational water loss through stomata is a key determinant of drought tolerance (Matsuda *et al*., 2016; Xiong *et al*., 2002). Regulation of stomatal movement in this regard has been an active research area for drought tolerance. For example, overexpression of *OsASR5*,* ONAC022*,* AtMYB61*,* BplMYB46* and *PtoMYB170* significantly enhanced drought tolerance in plants by regulation of stomatal closure under drought stress conditions (Guo *et al*., 2017; Hong *et al*., 2016; Li *et al*., 2017; Romano *et al*., 2012; Xu *et al*., 2017). Interestingly, overexpression of *AtMYB61*,* BplMYB46* and *PtoMYB170* also increased lignin biosynthesis.

In this study, we generated overexpression and knockdown transgenic lines of *OsTF1L*, a rice homeodomain‐leucine zipper transcription factor gene, and found that the overexpression lines enhanced drought tolerance and higher grain yield under both normal and drought conditions, whereas the knockdown lines remain susceptible to drought stress. *OsTF1L* overexpression elevates expression levels of lignin biosynthetic genes in shoots with a concomitant increase in the accumulation of lignin and enhances stomatal closure under drought stress conditions. This *OsTF1L*‐mediated drought tolerance mechanism increases our knowledge about interrelations among lignification, stomatal closure and drought tolerance in rice.

## Results

### Molecular characterization of *OsTF1L*

**Figure 1:**
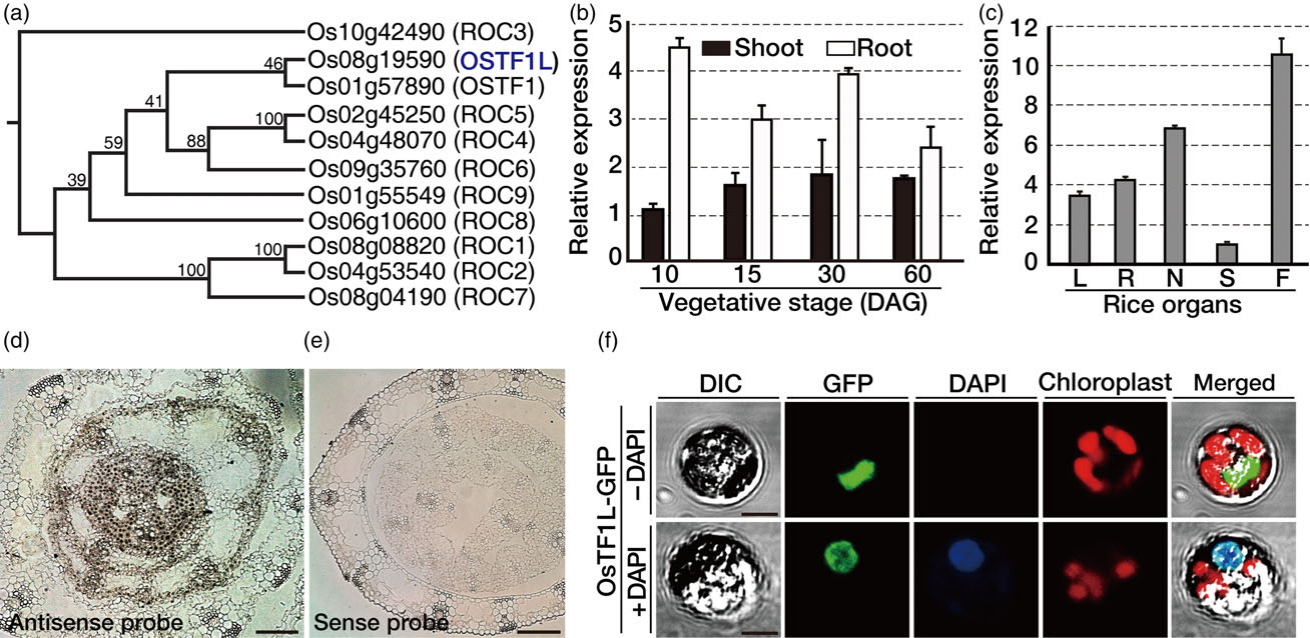
Expression patterns and subcellular localization of *OsTF1L*. (a) Phylogenetic tree created using the neighbour‐joining method in CLC sequence viewer using full‐length amino acid sequences of the rice HD‐ZIP IV proteins. Bootstrap support (100 repetitions) is shown for each node. (b, c) Quantitative RT‐PCR of *OsTF1L* in various tissues and at different growth stages. DAG, day after germination; L, leaf; R, root; N, node; S, sheath; F, flower. *Ubi1* (rice ubiquitin1) expression was used as an internal control. Data bars represent the mean ± SD of two biological replicates, each of which had three technical replicates. (d, e) *In situ* hybridization analysis of *OsTF1L* expression. Cross sections of shoot apex from 6‐day‐old seedling were probed with digoxigenin‐labelled antisense or sense strand of *OsTF1L* RNA. Scale bars, 100 μm. (f) Confocal images of OsTF1L‐GFP in rice protoplasts with DAPI staining. Scale bars, 5 μm.

Rice HD‐Zip class IV has 11 members, which consists of the conserved domains such as HD, Zip, steroidogenic acute regulatory protein‐related lipid transfer (START) and START‐associated domains (SAD) (Figures 1a and S1). Spatial‐temporal expression of *OsTF1L* (*Oryza sativa transcription factor 1‐like*), which belongs to the *OsTF1* subclade in a phylogenetic tree (Figure 1a), was determined by qRT‐PCR and *in situ* hybridization. Based on qRT‐PCR analyses, *OsTF1L* is expressed in all organs at various developmental stages (Figure 1b,c) and *in situ* hybridization analysis revealed that *OsTF1L* is expressed in the vasculature and outer cell layers, including epidermis (Figure 1d,e). To examine subcellular localization of OsTF1L, *OsTF1L* coding sequence was fused to the *GFP* (green fluorescent protein) reporter gene (Figure S2) under the control of the *GOS2* (rice eukaryotic translation initiation factor 1‐like gene) promoter and transformed into rice protoplasts (Figure 1f). Green fluorescent protein fluorescence was observed in the nucleus of protoplasts, as evidenced by co‐localization of the GFP signal with DAPI (4′, 6‐diamidino‐2‐phenylindole) nuclear DNA stain.

### *OsTF1L* modulates drought‐inducible, stomatal movement and lignin biosynthetic genes

**Figure 2:**
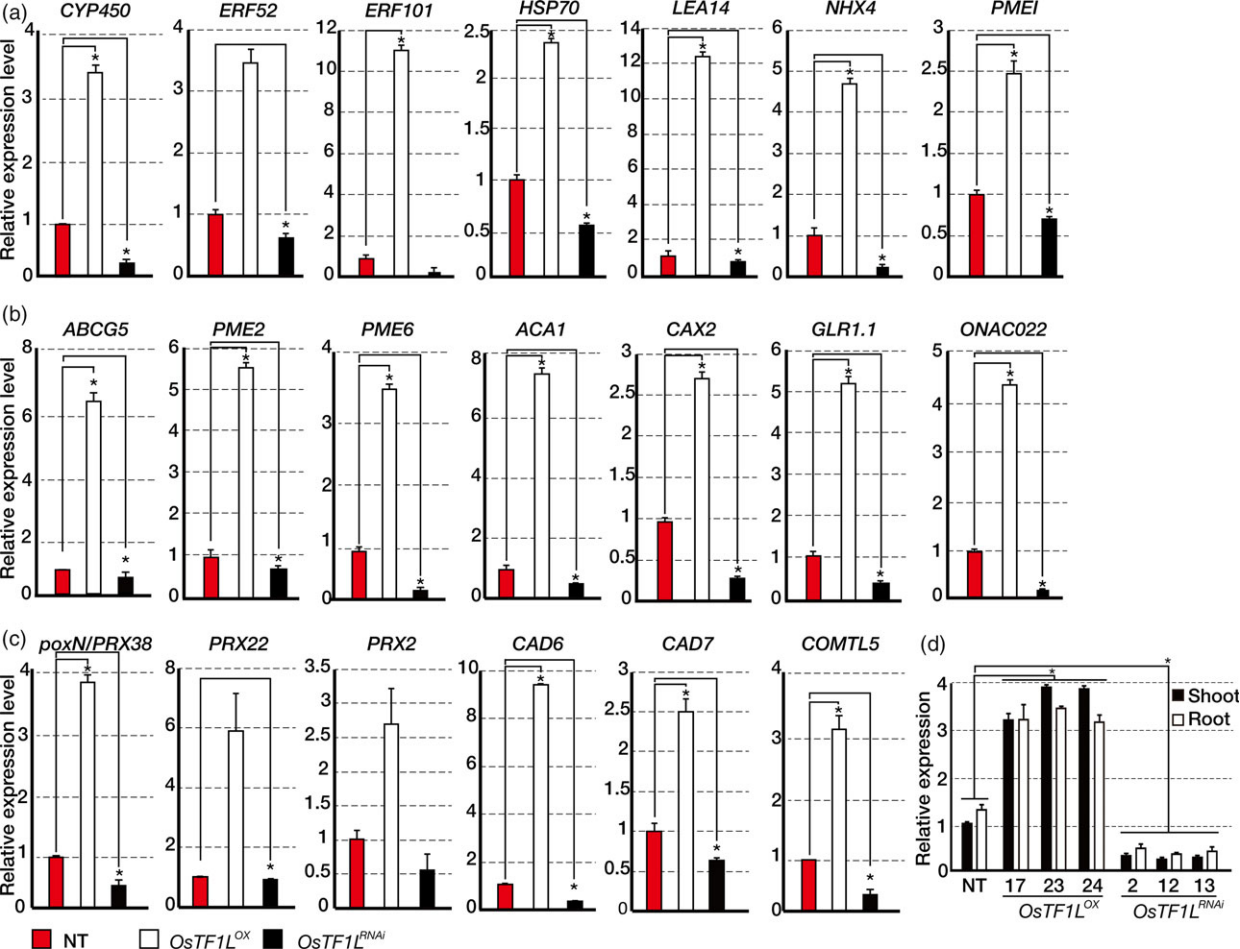
qRT‐PCR analysis showing the transcript levels of drought‐inducible genes (a), stomatal movement related genes (b) and lignin biosynthetic genes (c) in 1‐month‐old *OsTF1* *L* ^*OX*^ and *OsTF1* *L* ^*RNA*^ ^*i*^ shoots. *Ubi1* was used as the reference gene. Data are shown as the mean ± SD of three biological and two technical replicates. (d) qRT‐PCR analysis of *OsTF1L* expression in 5‐week‐old *OsTF1* *L* ^*OX*^ (#17, 23, 24), *OsTF1* *L* ^*RNA*^ ^*i*^ (#2, 12, 13) and NT plants. *Ubi* was used as an internal control. Data bars represent the mean ± SD of two biological replicates, each of which had three technical replicates. Asterisks indicate significant differences compared with NT (*P* < 0.05, one‐way ANOVA).

To uncover functional roles of the *OsTF1L*, we generated overexpression and RNAi transgenic rice plants. Overexpression (*OsTF1L* ^*OX*^) and RNAi knockdown (*OsTF1L* ^*RNAi*^) plants were generated using the *PGD1* (phosphogluconate dehydrogenase gene) and *GOS2* promoter, respectively, which are constitutively active throughout the whole plant body (Figure S2). Thirty independent lines for each type were initially generated, and lines with somaclonal variations were eliminated by successive field selection through T_3_ generation. We then selected three independent, homozygous lines for each of *OsTF1L* ^*OX*^ (#17, 23 and 24) and *OsTF1L* ^*RNAi*^ (#2, 12 and 13) for further analysis. The expression levels of *OsTF1L* were higher in the *OsTF1L* ^*OX*^ lines by 3.2‐ to fourfold and lower in the *OsTF1L* ^*RNAi*^ lines by 10‐ to 15‐fold, as compared to nontransgenic (NT) lines (Figure 2d). To identify *OsTF1L* target genes, we performed an RNA‐seq analysis with shoots from *OsTF1L* ^*OX*^ (#23) and NT control plants grown under normal conditions. A total of 1743 genes were expressed at higher levels (>2‐fold) in the *OsTF1L* ^*OX*^ shoots as compared to the NT controls, and they were categorized using GO analysis as follows: metabolic process (25%), response to stimuli (7%), catalytic activity (23%), and ion binding (15%; Figure S3a). We found that 59 drought‐inducible genes were up‐regulated in the *OsTF1L* ^*OX*^ shoots by filtering with a public database involving drought response genes (Chung *et al*., 2016; Table S2). These include *PECTIN METHYLESTERASE INHIBITOR PROTEIN* (*PMEI*), *LATE EMBRYOGENESIS ABUNDANT PROTEIN* (*LEA14*), *HEAT SHOCK PROTEIN* (*HSP70* and *90*), *Na* ^*+*^ */H* ^*+*^ *ANTIPORTER* (*NHX4*), *APELATA 2/ethylene response factor* (*ERF52* and *101*) and *CYTOCHROME P450* (*CYP450*). In addition, 25 genes related to stomatal movement were up‐regulated in the *OsTF1L* ^*OX*^ shoots. These include *ATP‐BINDING CASSETTE SUBFAMILY G PROTEIN* (*ABCG5*), *PECTIN METHYLESTERASE* (*PME2*,* 6*), *Ca* ^*2+*^ *P‐TYPE ATPASE* (*ACA1*), *VACUOLAR CATION/PROTON EXCHANGER* (*CAX2*), *GLUTAMATE RECEPTOR LIKE* (*GLR1.1*) and *NAC DOMAIN‐CONTAINING PROTEIN* (*NAC022*) (Hong *et al*., 2016; Matsuda *et al*., 2016; Pandey *et al*., 2007; Table S3). We also found that 29 genes involved in lignin biosynthesis are up‐regulated in the *OsTF1L* ^*OX*^ shoots. These include *PEROXIDASE* (*poxN/PRX38*,* PRX22* and *PRX2*), *CINNAMYL ALCOHOL DEHYDROGENASE* (*CAD6* and *7*), *CAFFEIC ACID O‐METHYLTRANSFERASE* (*COMTL5*) and *CINNAMATE‐4‐HYDROXYLASE* (*C4H*) (Barros *et al*., 2015; Hirano *et al*., 2012; Marjamaa *et al*., 2009; Wang *et al*., 2013; Xu *et al*., 2009; Table S4). Their increased and decreased levels of expression in the *OsTF1L* ^*OX*^ and the *OsTF1L* ^*RNAi*^ shoots, respectively, were validated by qRT‐PCR (Figure 2a–c). From this information, we concluded that *OsTF1L* modulates expression of drought‐inducible, stomatal movement and lignin biosynthetic genes.

### *OsTF1L* directly regulates five target genes

To examine the interaction between OsTF1L and promoters of the target genes, we performed chromatin immunoprecipitation (ChIP)‐seq analysis on the *OsTF1L* ^*OX*^ (*myc*‐tagged *OsTF1L* overexpressor) and NT shoots. Two different antibodies, anti‐myc and anti‐RNA Pol II (Figure S3b), were used to probe interacting loci. Genomewide ChIP‐seq analysis led us to identify 345 putative OsTF1L binding sites in the promoter regions (defined here as within a 3‐kb region upstream from the transcription start site) of 514 genes. Of these, 336 genes were enriched by more than twofold compared to the NT controls in the RNA Pol II ChIP‐seq analysis. Thus, by filtering with RNA Pol II ChIP results, we could select interacting loci that are transcriptionally active. Finally, by cross‐referencing of the selected 336 genes with the RNA‐seq results (>2‐fold; Figure S3c), we identified five putative direct targets of *OsTF1L*:* poxN/PRX38*,* Nodulin protein*,* AAA‐type ATPase*,* DHHC4* and *CASPL5B1* (Table 1). The genes were down‐ or up‐regulated in *OsTF1L* ^*RNAi*^ and *OsTF1L* ^*OX*^ shoots, respectively (Figure S3d). The *AAA‐type ATPase* gene was previously reported to be involved in drought tolerance, and other four genes play roles in lignification (Ito *et al*., 2000; Qi *et al*., 2013; Ranocha *et al*., 2010; Roppolo *et al*., 2011, 2014; Xia *et al*., 2013; Zhou *et al*., 2013). These results suggest that OsTF1L binds directly to promoters of the target genes and regulates their expression to enhance drought tolerance and lignin biosynthesis in rice.

**Table 1:** List of putative target genes directly regulated by *OsTF1L* based on the chromatin immunoprecipitation (ChIP)‐seq analysis using RNA Pol II ChIP‐seq and the RNA‐seq data

Binding locus	Gene ID	Description	ChIP‐seq					RNA‐seq			
			RPM (Normalization)			Log2 ratio		PKM (Normalization)			Log2 ratio
			NT‐total	OX‐myc	OX‐Pol II	OX myc/NT‐total	OX‐Pol II/NT‐total	NT	OX	Fold	
chr03‐7132344‐7133928	Os03g0235000	*poxN/PRX38*	7.58	29.64	17.20	1.84	1.08	0.09	0.35	3.78	1.92
chr01‐40572571‐40574276	Os01g0925300	*DHHC4*	11.57	47.32	28.60	1.94	1.24	0.14	0.38	2.64	1.40
chr01‐36403281‐36404182	Os01g0847300	*CASPL5B1*	1.20	6.59	5.91	1.79	1.65	0.84	2.14	2.55	1.35
chr03‐33479057‐33480620	Os03g0802500	*AAA‐type ATPase*	21.72	65.00	47.07	1.54	1.08	10.46	38.22	3.65	1.87
chr12‐27330005‐27333766	Os12g0637800	Nodulin protein	0.06	5.55	3.27	2.63	2.01	0.07	0.19	2.60	1.38

**Figure 3:**
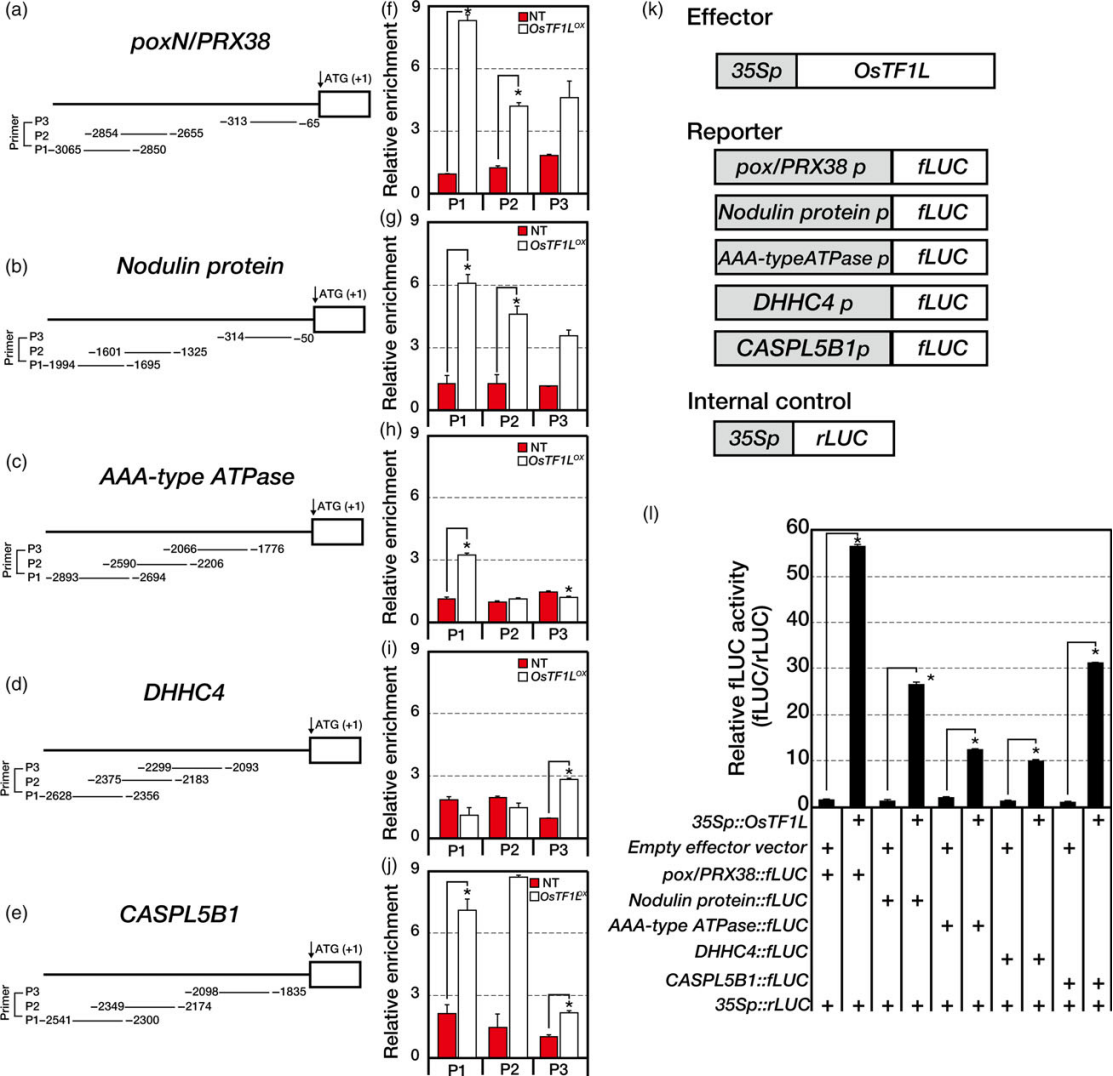
ChIP‐qPCR and transient protoplast assays for *OsTF1L* interacting with promoters of the selected genes in ChIP‐seq and RNA‐seq analysis. (a–j) Two‐week‐old *OsTF1* *L* ^*OX*^ and NT shoots were used in the ChIP‐qPCR experiments with an anti‐myc antibody. (a–e) Promoter regions showing three PCR‐amplified regions (P1–P3). (f–j) ChIP‐qPCR data show each PCR amplification region of each gene. The relative enrichment was normalized with total input. Data are shown as the mean ± SD of three independent experiments. (k, l) Transient protoplast expression assay using a dual‐luciferase reporter system. (k) Schematic diagram of the effector, internal control and five reporter constructs. (l) Relative fLUC (fLUC/rLUC) activity in rice protoplasts. Data are shown as the mean ± SD of three independent experiments. Asterisks indicate significant differences compared with NT (*P* < 0.05, Student's *t*‐test).

The target genes were found to have potential HD‐binding *cis*‐elements AAATTAAA, AAATTAGT, TAAATGTA, CAATGATTG and TGCATTTA (Xu *et al*., 2014) in their promoters. The interactions between OsTF1L and promoter regions of the five target genes were confirmed by ChIP‐qPCR analysis (Figure 3a–j). We further validated the binding activity of OsTF1L to the five target genes by a transactivation assay. Promoters of the five genes were fused to a firefly luciferase (fLUC) reporter and co‐transfected into rice protoplasts with either the *35S::OsTF1L* construct or an empty vector (Figure 3k). As a result, expression of the five reporter constructs was significantly elevated in the presence of the *35S::OsTF1L* construct, but not in the presence of the empty vector (Figure 3l). These results suggest that the OsTF1L binds and activates five direct target genes involved in drought tolerance and lignification.

### *OsTF1L* overexpression in rice plants confers drought tolerance at the vegetative stage

**Figure 4:**
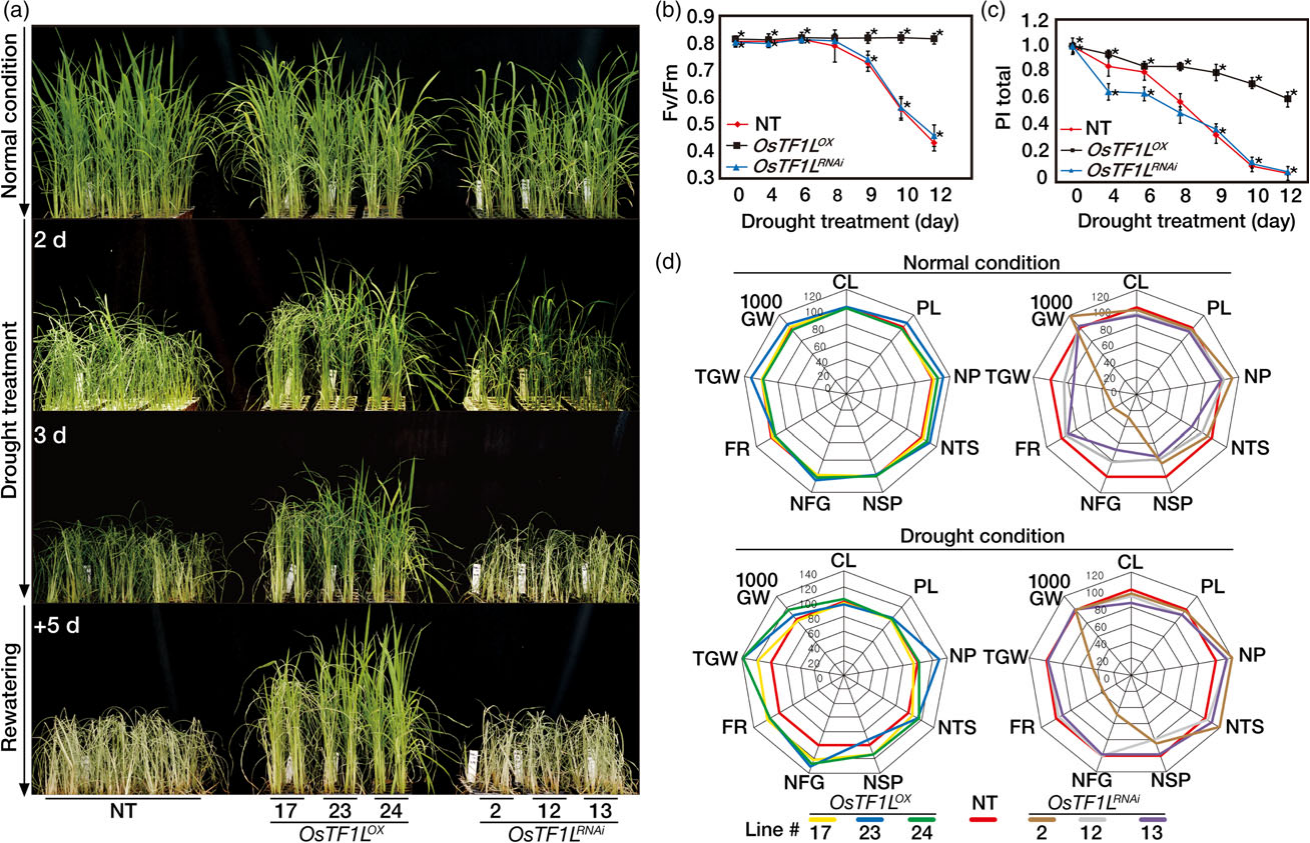
*OsTF1L* overexpression in rice confers drought tolerance. (a) Phenotypes of *OsTF1* *L* ^*OX*^ and *OsTF1* *L* ^*RNA*^ ^*i*^ transgenic rice plants under drought stress at the vegetative stage. Three independent homozygous *OsTF1* *L* ^*OX*^ and *OsTF1* *L* ^*RNA*^ ^*i*^ T_3_ lines and nontransgenic (NT) control plants were grown in soil for 5 weeks and exposed to drought for 3 days, followed by re‐watering. (b) Chlorophyll fluorescence (*F*_v_ */F*_m_) of *OsTF1* *L* ^*OX*^ and *OsTF1* *L* ^*RNA*^ ^*i*^ transgenic rice plants and nontransgenic (NT) plants during a 12‐day drought treatment. *F*_v_ */F*_m_ values were measured in the dark to ensure sufficient dark adaptation. Data are shown as the mean ± SD (*n* = 30). (c) Performance index (PI_total_) of *OsTF1* *L* ^*OX*^ and *OsTF1* *L* ^*RNA*^ ^*i*^ transgenic plants and NT plants during drought conditions for 12 days. Data are shown as the mean ± SD (*n* = 30). (d) Agronomic traits of *OsTF1* *L* ^*OX*^ and *OsTF1* *L* ^*RNA*^ ^*i*^ transgenic rice plants grown in field under both normal and drought conditions. Spider plots of yield parameters with three independent homozygous T_4_ lines of *OsTF1* *L* ^*OX*^ and two independent homozygous T_3_ lines of *OsTF1* *L* ^*RNA*^ ^*i*^ under normal and drought conditions, respectively. Each data point shows a percentage of the means values (*n* = 30) listed in Table 2. Mean values from NT controls were set at 100% as a reference. CL, culm length; PL, panicle length; NP, number of panicles per hill; NTS, number of total spikelets; NSP, number of spikelets per panicle; NFG, number of filled grains; FR, filling rate; TGW; total grain weight; 1000 GW, 1000 grain weight.

As a number of drought‐inducible, stomatal movement and lignin biosynthetic genes were differentially expressed by *OsTF1L* overexpression, we first tested drought tolerance of the *OsTF1L* ^*OX*^ and *OsTF1L* ^*RNAi*^ lines after exposed to drought stress. Five‐week‐old plants were exposed to drought conditions for 3 days by withholding irrigation, where soil moisture content was uniformly decreased over time and reached approximately 10% of soil moisture content at 3 days after the onset of the drought treatments (Figure S4). The *OsTF1L* ^*OX*^ plants showed delayed visual symptoms of drought‐induced damage, such as leaf rolling and wilting, as compared with the NT and the *OsTF1L* ^*RNAi*^ plants. After rehydration, the *OsTF1L* ^*OX*^ plants rapidly recovered, whereas the NT and *OsTF1L* ^*RNAi*^ plants did not (Figure 4a). The *OsTF1L* ^*RNAi*^ plants remained as sensitive as the NT plants to the drought stress. Collectively, these results suggested that *OsTF1L* overexpression confers drought tolerance during vegetative growth.

To validate the drought tolerance of the transgenic plants under delayed drought conditions, 6‐week‐old plants were transplanted to big pots (two plants per line) and exposed to drought stress by withholding irrigation for 12 days (Figure S5a). Soil moisture contents consistently decreased in the pots containing each of the different genotypes (Figure S5b). Physiological changes were measured using the JIP test, which reflects the activity of the whole photosynthetic machinery affected by drought stress: *F*_v_/*F*_m_ (*F*_v_: variable fluorescence and *F*_m_: maximum fluorescence) value for photochemical efficiency of photosystem (PS) II, and the PI_total_ (performance index) value for photochemical efficiency of PS I and II (Redillas *et al*., 2011; Strasser *et al*., 2004). *F*_v_/*F*_m_ values of NT and *OsTF1L* ^*RNAi*^ plants were rapidly declined during 8 days after drought treatments, whereas those of *OsTF1L* ^*OX*^ plants remained unchanged (Figure 4b). In addition, the PI_total_ values of the *OsTF1L* ^*OX*^ plants were higher than those of the NT and *OsTF1L* ^*RNAi*^ plants during the drought treatments, indicating that the photochemical efficiencies of PS I and PS II were less damaged by drought in the *OsTF1L* ^*OX*^ lines than in the NT and *OsTF1L* ^*RNAi*^ plants. PI_total_ values of the *OsTF1L* ^*RNAi*^ plants decreased sharply up to 8 days after the beginning of the treatment as compared with the NT plants, indicating that the photochemical efficiency of PS I was more perturbed by drought in the *OsTF1L* ^*RNAi*^ plants than in the NT plants (Figure 4c). These opposite physiological phenotypes in the overexpression and RNAi lines are consistent with a role of *OsTF1L* in promoting drought tolerance.

### *OsTF1L* overexpression significantly increases grain yield under field‐drought conditions

We evaluated yield parameters of the *OsTF1L* ^*OX*^ and the *OsTF1L* ^*RNAi*^ plants grown in a paddy field under normal and drought stress conditions. The yield parameters were scored from 30 plants per transgenic line, with three replicates (Table 2 and Figure 4d). Under normal growth conditions, the yield parameters of the *OsTF1L* ^*OX*^ plants including the grain‐filling rate and total grain weight were similar or slightly greater than the NT control plants, whereas those of the *OsTF1L* ^*RNAi*^ plants were lower. Interestingly, under drought stress conditions, the yield parameters of all the *OsTF1L* ^*OX*^ plants were significantly higher than those of the NT controls, while those of *OsTF1L* ^*RNAi*^ plants were lower. Thus, overexpression of *OsTF1L* enhanced drought tolerance and increased grain yield at the reproductive growth stage, in addition to increasing drought tolerance at the vegetative stage.

**Table 2:** Agronomic traits of *OsTF1L* ^*OX*^ and *OsTF1L* ^*RNAi*^ transgenic rice plants under normal conditions

	Culm length (cm)	Panicle Length (cm)	No. of Panicles (/hill)	No. of total spikelets (/hill)	No. of spikelets (/panicle)	No. of filled grains	Filling rate (%)	Total Grain weight (g)	1000 GW (g)
Normal condition									
NT (Ilmi)	68.71	20.60	13.09	1484.30	114.21	1372.04	92.35	37.87	27.69
*OsTF1L* ^*OX*^ *‐17*	67.53	20.21	13.33	1505.94	114.94	1358.44	90.4*	37.55	27.82
*P* value	0.683	0.813	0.151	0.604	0.185	0.777	0.033	0.816	0.853
*OsTF1L* ^*OX*^ *‐23*	69.60	22.07**	14.7	1647.37	112.64	1449.85	88.25**	42.17	29.28**
*P* value	0.181	0.003	0.123	0.250	0.471	0.710	0.000	0.186	0.000
*OsTF1L* ^*OX*^ *‐24*	68.23**	20.46	13.84**	1590.79**	114.57	1396.58**	88.18**	37.24**	26.94*
*P* value	0.001	0.328	0.000	0.000	0.135	0.000	0.000	0.000	0.040
*OsTF1L* ^*RNAi*^ *‐2*	66.34*	19.99*	15.00**	1439.87**	97.13**	386.53**	27.28**	14.15**	37.01**
*P* value	0.011	0.072	0.004	0.009	0.000	0.000	0.000	0.000	0.000
*OsTF1L* ^*RNAi*^ *‐12*	63.60**	19.91	13.37	1302.56**	91.04**	1124.63**	86.28**	31.76**	28.35*
*P* value	0.000	0.077	0.055	0.004	0.000	0.000	0.000	0.000	0.021
*OsTF1L* ^*RNAi*^ *‐13*	63.14**	19.36	12.95	1100.75**	86.07**	929.70**	84.02**	28.11**	28.56**
*P* value	0.000	0.121	0.324	0.000	0.000	0.000	0.000	0.000	0.000
Drought condition									
NT (Ilmi)	56.44	17.85	11.53	759.94	67.00	503.94	64.71	12.07	24.11
*OsTF1L* ^*OX*^ *‐17*	55.23	17.57	10.87	810.27	76.53	613.33	77.61	14.29	23.48
*P* value	0.576	0.297	0.405	0.654	0.753	0.096	0.100	0.145	0.072
*OsTF1L* ^*OX*^ *‐23*	54.50*	18.12	14.92*	872.46	61.30	661.69**	74.75	16.62**	25.54*
*P* value	0.014	0.368	0.041	0.051	0.648	0.008	0.084	0.006	0.028
*OsTF1L* ^*OX*^ *‐24*	58.00	17.70	11.80	875.80**	75.73	640.00	74.10	16.61*	27.64
*P* value	0.712	0.234	0.064	0.008	0.196	0.160	0.135	0.027	0.632
*OsTF1L* ^*RNAi*^ *‐2*	53.35	17.38	17.76*	1089.35**	64.74*	300.35	29.20*	6.49	23.70
*P* value	0.135	0.093	0.012	0.001	0.048	0.137	0.014	0.069	0.716
*OsTF1L* ^*RNAi*^ *‐12*	52.04	16.56	13.15**	787.54	53.26	497.23	63.08	11.79	24.35
*P* value	0.059	0.098	0.001	0.536	0.050	0.132	0.155	0.278	0.182
*OsTF1L* ^*RNAi*^ *‐13*	47.72**	16.41	12.94*	829.44	65.28	489.69	59.19	11.89	24.43
*P* value	0.000	0.081	0.033	0.214	0.436	0.117	0.191	0.271	0.073

Each data value represents the mean (*n* = 30) for *OsTF1LOX*,* OsTF1LRNAi* and nontransgenic (NT) control rice plants (*P < 0.05 and **P < 0.01, one‐way ANOVA).

### Overexpression of *OsTF1L* increases stomatal closure

**Figure 5:**
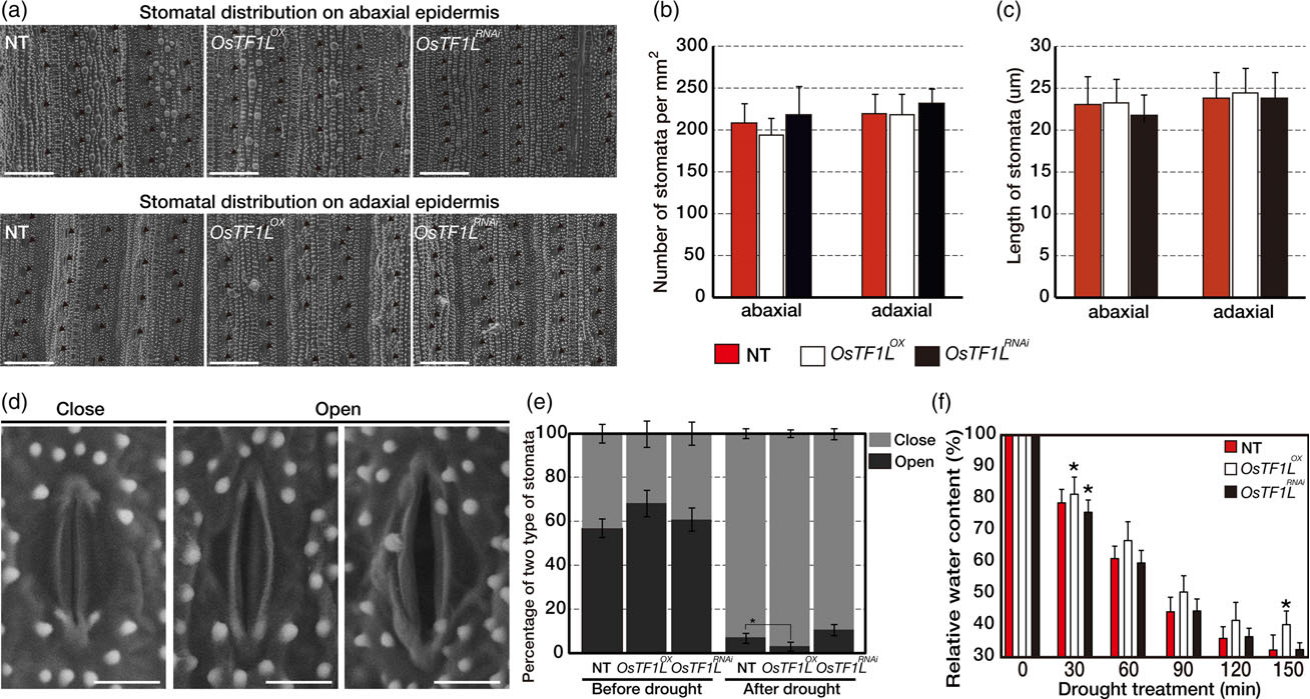
Overexpression of *OsTF1L* Increasing Stomatal Closure. (a) Scanning electron microscopy images (600×) of the abaxial and adaxial leaf epidermis, where stomata are marked by black arrowheads. Scale bar represents 100 μm. (b) Average stomata numbers per square millimetre calculated from 10 plants for each line. Values are mean ± SD. (c) Comparison of the stomata length between NT and transgenic plants. Twenty stomata were measured for each plant. Ten plants were used for each line. Values are mean ± SD. (d) Scanning electron microscopy images (2000×) of two levels of stomata opening. Scale bar represents 10 μm. (e) The percentage of two levels of stomatal apertures in the leaves of *OsTF1* *L* ^*OX*^ ,* OsTF1* *L* ^*RNA*^ ^*i*^ and NT plants under normal and drought stress conditions. Twenty stomata were measured for each plant. Ten plants were used for each line. Values are mean ± SD. Asterisks indicate significant differences compared with NT (*P* < 0.05, one‐way ANOVA). (f) Relative water content of detached leaves. For each replicate, fully expanded ten leaves of 3‐month‐old mature plants were used for each line. Data are shown as the mean ± SD of three independent lines and two experimental replicates. Asterisks indicate significant differences compared with NT (*P* < 0.05, one‐way ANOVA).

As the RNA‐seq data showed up‐regulation of genes involved in stomatal movement, we further examined the stomatal status of *OsTF1L* ^*OX*^, *OsTF1L* ^*RNAi*^ and NT plants under normal and drought‐treated conditions. Leaf surfaces were observed using SEM to determine the numbers and size of stomata (Figure 5a). As shown in Figure 5b,c, there is no significant difference in the average number of stomata per square millimetre and length of stomatal cell on both the abaxial and adaxial surfaces of the *OsTF1L* ^*OX*^ *, OsTF1L* ^*RNAi*^ and NT plants. We next examined stomatal opening and closing in the *OsTF1L* ^*OX*^ *, OsTF1L* ^*RNAi*^ and NT leaves. As the stomata could be classified as the close and open (Figure 5d), the stomatal apertures were measured both before and after drought treatments (Figure 5e). Before drought treatment, NT leaves showed 43.0% close and 57.0% open stomata, whereas *OsTF1L* ^*OX*^ and *OsTF1L* ^*RNAi*^ leaves displayed 31.8% and 39.1% close and 68.2% and 60.9% open stomata, respectively. After drought treatment, NT leaves showed 92.8% close and 7.2% open stomata. However, *OsTF1L* ^*OX*^ and *OsTF1L* ^*RNAi*^ leaves were observed by 96.7% and 89.3% close and 3.3% and 10.7% open stomata, respectively. These results indicate that stomatal closure in the *OsTF1L* ^*OX*^ was more than in the *OsTF1L* ^*RNAi*^ and NT plants under drought conditions. Finally, we tested whether water loss from *OsTF1L* ^*OX*^ leaves was slower than that from NT and *OsTF1L* ^*RNAi*^ leaves under drought conditions (Figure 5f). *OsTF1L* ^*OX*^ leaves slowly lost water rather than NT and *OsTF1L* ^*RNAi*^ leaves under drought conditions. These data indicate that drought tolerance phenotype of the *OsTF1L* ^*OX*^ plants is caused in part by acceleration of stomatal closure.

### Overexpression of *OsTF1L* significantly increases lignin accumulation

**Figure 6:**
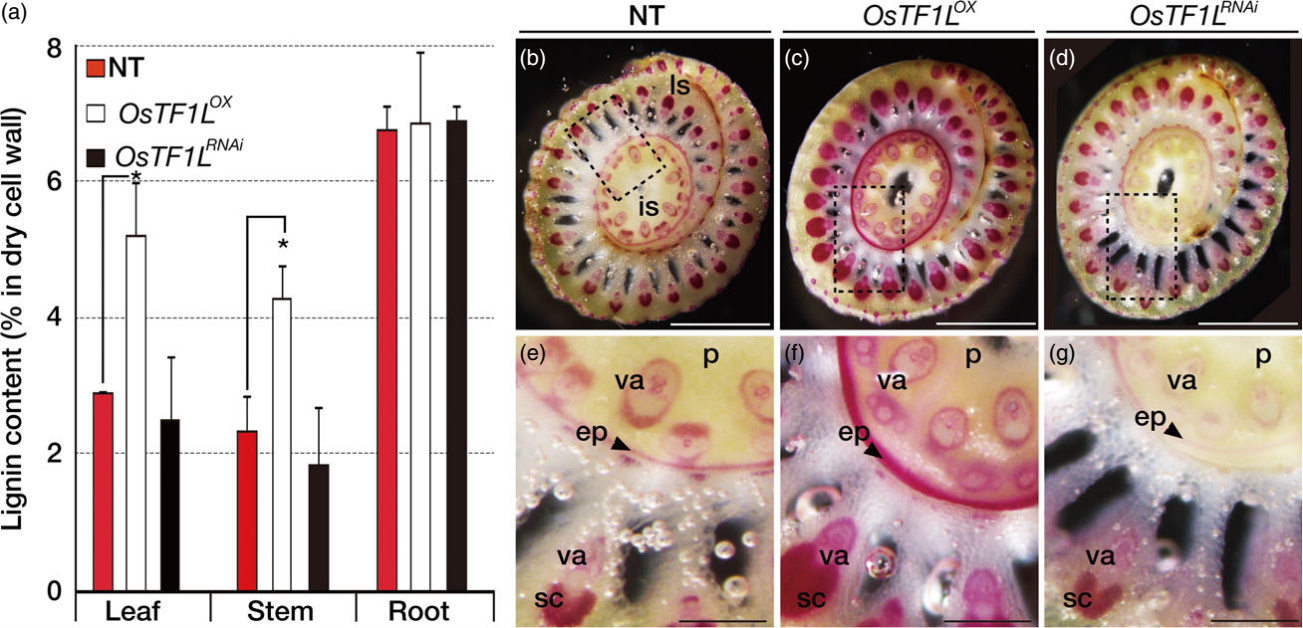
Accumulation of lignin in *OsTF1* *L* ^*OX*^ ,* OsTF1* *L* ^*RNA*^ ^*i*^ transgenic rice and control plants. (a) Lignin contents in 2‐month‐old *OsTF1* *L* ^*OX*^ ,* OsTF1* *L* ^*RNA*^ ^*i*^ and nontransgenic (NT) rice plants. Data bars represent the mean ± SD of three biological replicates (*n* = 3), each of which had two technical replicates. Asterisks indicate significant differences compared with NT (*P* < 0.05, one‐way ANOVA). (b–g) Transverse hand sections of phloroglucinol‐HCl stained 3‐month‐old mature shoots of NT (left), *OsTF1* *L* ^*OX*^ (middle) and *OsTF1* *L* ^*RNA*^ ^*i*^ (right) plants. (e–g) are enlarged views of the dotted line boxes in (b), (c) and (d), respectively. ls, leaf sheath; is, inflorescence stem; p, pith; va, vascular bundles; ep, epidermis; sc, sclerenchyma tissues.

Up‐regulation of lignin biosynthetic genes by the *OsTF1L* overexpression led us to measure lignin levels in the *OsTF1L* ^*OX*^, *OsTF1L* ^*RNAi*^ and NT plants (Figure 6a and Table 3). Lignin levels were higher by 1.8‐fold in leaves and stems of the *OsTF1L* ^*OX*^ plants and lower in the corresponding tissues of *OsTF1L* ^*RNAi*^ plants than the NT control plants. In contrast, lignin levels in roots of the *OsTF1L* ^*OX*^ *, OsTF1L* ^*RNAi*^ and NT plants remained unchanged. Lignin‐specific (Phloroglucinol‐HCL) staining of those rice shoots confirmed the increased and decreased levels of lignin in the *OsTF1L* ^*OX*^ and *OsTF1L* ^*RNAi*^ plants (Figure 6b–g). In particular, the epidermis of the inflorescence stem, as well as the sclerenchyma tissues and vasculature of the leaf sheath of the *OsTF1L* ^*OX*^ plants, was highly lignified, whereas the corresponding tissues of the *OsTF1L* ^*RNAi*^ and NT plants remained less lignified. *In situ* hybridization data that *OsTF1L* is mainly expressed in the outer cell layers, including the epidermis, and the vasculature of the sheaths (Figure 1d) coincides with areas of increased lignification. Taken together, our results indicate that *OsTF1L* modulates lignification via up‐regulation of lignin biosynthetic genes in rice, which contributes to the drought tolerance phenotype.

**Table 3:** Lignin content of leaf, stem and root cell walls of 1‐month‐old plants

Sample (mg/g cell wall)	Leaf	Stem	Root
NT	0.58 ± 0.00	0.47 ± 0.10	1.35 ± 0.07
*OsTF1L* ^*OX*^	1.04 ± 0.15	0.85 ± 0.10	1.37 ± 0.21
*OsTF1L* ^*RNAi*^	0.50 ± 0.19	0.37 ± 0.17	1.38 ± 0.04

Each data value represents the mean ± SD (*n* = 30) of 10 plants and three technical replicates.

## Discussion

In this study, we found that overexpression of *OsTF1L* improves drought tolerance and grain yield of rice, providing a valuable candidate for crop biotechnology. The *OsTF1L* ^*OX*^ plants showed reduced and delayed drought‐induced damage, compared with the control plants and recovered more rapidly after rehydration. They also showed decreased water loss and maintained normal photosynthesis efficiency under drought conditions. Although drought stress at the vegetative stage can affect rice growth and development, drought tolerance at the reproductive stages is more important for grain productivity (Ambavaram *et al*., 2014; Nuccio *et al*., 2015; Xiao *et al*., 2009; Yu *et al*., 2013). We observed that yield parameters of the *OsTF1L* ^*OX*^ plants were significantly higher under field‐drought conditions, and saw no evidence of growth retardation or yield trade‐off. In contrast, the *OsTF1L* ^*RNAi*^ plants were drought‐sensitive and had lower yield parameters than the NT control plants under both drought and normal field conditions.

Lignin is a biopolymer, composed of p‐coumaryl, coniferyl and sinapyl alcohol monomers, which makes cell walls stiffer. This study identified that *OsTF1L* overexpression increased the degree of shoot lignification. Specifically, lignin levels in the *OsTF1L* ^*OX*^ plants were higher in the epidermis of the inflorescence stem, sclerenchyma tissues and vasculature of the leaf sheath, but were lower in the same tissues of the *OsTF1* ^*RNAi*^ than in the control plants. *In situ* hybridizations demonstrated that the *OsTF1L* is expressed in the outer cell layers including epidermis and vasculature of sheaths that coincides with areas of increased lignification in the *OsTF1L* ^*OX*^ plants. Despite the whole plant body overexpression of *OsTF1L*, enhanced lignification of the *OsTF1L* ^*OX*^ plants occurred only in the typically lignified tissues, rather than being ectopically expressed throughout the plant. These observations were supported by our RNA‐seq and qRT‐PCR analyses that lignin biosynthetic genes were up‐ and down‐regulated in the *OsTF1L* ^*OX*^ and the *OsTF1L* ^*RNAi*^ shoots, respectively. However, expression of *PRX 2*,* CAD6*,* CAD7* and *COMTL5* was not changed in *OsTF1L* ^*OX*^ roots as compared with NT (Figure S6). Several independent lines of evidence support the lignin‐mediated drought‐tolerant phenotype. Drought‐tolerant maize inbred lines under drought stress conditions display higher lignification than drought‐sensitive inbred lines (Hu *et al*., 2009). Also, the *curled leaf and dwarf 1* (*cd1*) mutant exhibit significant decrease in lignin contents in secondary cell walls causing water deficits in *cd1* leaves and resulting to reduced drought tolerance (Li *et al*., 2017). In addition, reduced lignification in plants increases their xylem vulnerability to embolism (Voelker *et al*., 2011). Cavitation occurs when the xylem conduits break and a void is created. The void is initially filled with water vapour, but air can diffuse into it, forming an embolism (Stiller *et al*., 2005). Resistance to cavitation is known to be directly related to drought tolerance (Urli *et al*., 2013). Likewise, the *OsTF1L*‐mediated lignification of shoots might contribute to the improved drought tolerance in *OsTF1L* ^*OX*^ plants.

In addition to lignin biosynthetic genes, many drought‐inducible and stomatal movement genes were up‐regulated in *OsTF1L* ^*OX*^ plants (Tables S2 and S3). The drought‐inducible genes include *PMEI*, a gene for an enzyme that inhibits demethylesterification of pectin, which improves drought tolerance when overexpressed (An *et al*., 2008). *LEA*,* HSP*,* NHX*,* CYP450* and *AP2/ERF* are among the up‐regulated drought‐inducible genes, which were reported to improve drought tolerance when overexpressed (Brini *et al*., 2007; Oh *et al*., 2009; Sato and Yokoya, 2008; Tamiru *et al*., 2015; Xu *et al*., 1996). Furthermore, up‐regulation of stomatal movement genes contributes to drought tolerance. The *reduced culm number 1* (*rcn1*) mutant encoding *OsABCG5* shows rapid water loss more than the wild‐type plants by deficiency of stomatal closure during drought stress (Matsuda *et al*., 2016). The rice NAC gene, *ONAC022*, was also identified as a positive role in drought tolerance through modulating a stomatal closure (Hong *et al*., 2016). The up‐regulation of stomatal movement genes is consistent with our observation that *OsTF1L* ^*OX*^ plants showed high ratio of the stomatal closure under drought conditions. By increased stomatal closure, *OsTF1L* ^*OX*^ plants gave rise to reduced rates of water loss under drought conditions. Taken together, these data demonstrate that *OsTF1L* modulates multiple molecular mechanisms for drought tolerance including stomatal movement and lignin biosynthesis.

To further understand the *OsTF1L*‐mediated drought tolerance mechanisms in rice, we identified five direct target genes of *OsTF1L* by the ChIP‐seq and RNA‐seq analysis: *poxN/PRX38*,* DHHC4*,* Nodulin protein*,* CASPL5B1* and *AAA‐type ATPase*. These genes were up‐regulated during transient expression of *OsTF1L* in rice protoplasts and reported to have roles in lignin biosynthesis and drought tolerance. The *poxN/PRX38* is responsible for the last step of lignin biosynthesis (Ito *et al*., 2000). The mutant in the *AtPAT10* (*Protein S‐acyltransferases 10*), a member of the DHHC family, was shown to result in reduction in lignified xylem (Qi *et al*., 2013; Zhou *et al*., 2013). The *WAT1*, an *Arabidopsis* homolog of our rice *Nodulin* gene, has been shown to reduce lignification in secondary cell wall (Ranocha *et al*., 2010). In addition, CASPARIAN STRIP MEMBRANE DOMAIN PROTEINS (CASPs) are known to mediate the deposition of Casparian strips in the endodermis by recruiting the lignin polymerization machinery (Roppolo *et al*., 2011, 2014). This raises a possibility that *CASPL5B1*, a member of the CASP‐like protein family, might have some roles in lignin biosynthesis. Additionally, overexpression of *SKD1*, a member of the AAA‐type ATPase proteins, enhanced tolerance to drought and reduced accumulations of reactive oxygen species (ROS; Xia *et al*., 2013).

In conclusion, *OsTF1L* has a biological role for drought tolerance by modulating drought‐inducible, stomatal movement and lignin biosynthetic genes. We found that overexpression of the *OsTF1L* gene increases drought tolerance capacity in rice and OsTF1L binds to promoters of five direct target genes, which are associated with lignin biosynthesis and drought tolerance. This study provides novel insights into the function of the HD‐Zip class IV family in rice, further indicating its potential importance for crop biotechnology.

## Experimental procedures

### Plasmid construction and *Agrobacterium*‐mediated rice transformation

For the overexpression of genes in rice, the full‐length *OsTF1L* cDNA (AK100441; Os08g0292000) was amplified by RT‐PCR according to the manufacturer's instructions (Promega, Madison, WI). The coding sequence was inserted into the pE3c vector, which is flanked with a 6 × myc tag coding sequence (Dubin *et al*., 2008). Finally, the OsTF1L‐6 × myc sequence from the pE3c‐OsTF1L plasmid was subcloned into the p700 rice transformation vector (Lee *et al*., 2016) carrying the *PGD1* promoter (Park *et al*., 2010) using the Gateway system (Invitrogen, Carlsbad, CA). This construct was named *OsTF1L* ^*OX*^ and was used for constitutive overexpression (Figure S2). For the suppression of *OsTF1L* expression in rice, 321 bp from the *OsTF1L* coding region (2103–2423 bp upstream of start codon ATG) was inserted into two sites of the p700‐GOS2‐RNAi vector (Lee *et al*., 2016), separated by a β‐glucuronidase (GUS) reporter gene sequence (Figure S2). For transient expression of OsTF1L*‐*GFP in rice protoplasts, the *OsTF1L* coding sequence without the stop codon was inserted into the p600 rice transformation vector (Lee *et al*., 2015) between the *GOS2* promoter (de Pater *et al*., 1992) and the GFP coding sequence. The vectors were introduced into wild‐type rice (*Oryza sativa* L. var. Japonica cv. Illmi) using the *A. tumefaciens* (strain LBA4404)‐mediated co‐cultivation (Jang *et al*., 1999). For each construct, thirty independent lines were initially generated. Transgene expression levels in T_0_ transgenic lines were determined using the qRT‐PCR and selected the best five overexpressing lines for *OsTF1L* ^*OX*^ or five down‐regulating lines for *OsTF1L* ^*RNAi*^. At T_1_ generation, homozygous lines were selected through growth in phosphinothricin‐containing MS media. Transgenic lines with somaclonal variations were eliminated by successive field selection through the T_3_ generation. Finally, we obtained three independent homozygous lines for each of *OsTF1L* ^*OX*^ (#17, 23 and 24) and *OsTF1L* ^*RNAi*^ (#2, 12 and 13) and used for further analysis.

### qRT‐ PCR analysis

For gene expression analysis, cDNA was synthesized with oligo‐dt primers using a first‐strand cDNA synthesis kit (Fermentas, Burlington, ON). qRT‐PCR was carried out using 2 × qRT‐PCR Pre‐mix with 20 × EvaGreen™ (SolGent, Seoul, Korea). The amplification reactions were performed at 95°C for 10 min, followed by 40 cycles of 95°C for 20 s, 58°C for 40 s, 72°C for 20 s, in a 20 μL mix containing 1 μL of 20 × EvaGreen™, 0.25 μM primers and 10 ng cDNA. qRT‐PCR analysis was performed using a Stratagene Mx3000p instrument and Mx3000p software, v2.02 (Stratagene, La Jolla, CA). The rice *ubiquitin1* (AK121590) transcript was used as a normalization control, and three biological and two technical replicates were analysed for all quantitative experiments. To investigate the spatial and temporal expression patterns of *OsTF1L*, total RNA was extracted from shoots and roots of 10‐, 15‐, 30‐ and 60‐day‐old NT rice plants at the vegetative stages, and from leaves, roots, node, stems, sheathes and flowers at the reproductive stages. To measure *OsTF1L* expression levels in *OsTF1L*‐overexpressing and knockdown plants, and to validate the RNA‐seq data, total RNA samples were extracted from the shoots of 2‐week‐old transgenic and NT rice seedlings. All primer pairs are listed inTable  S1.

### Measurement of chlorophyll fluorescence

To measure *F*_v_/*F*_m_ and PI_total_ values, chlorophyll fluorescence was measured using the Handy‐PEA fluorimeter (Plant Efficiency Analyzer; Hansatech Instruments, King's Lynn, Norfolk, UK) under dark conditions to ensure sufficient dark adaptation (at least 1 h; Redillas *et al*., 2011). *OsTF1L* ^*OX*^, *OsTF1L* ^*RNAi*^ and NT plants were grown in a glasshouse at 28–30°C for 6 weeks. Ten leaves from each plant were analysed and values were calculated using Handy PEA software (version 1.31) and Biolyzer 4HP software (v4.0.30.03.02).

### Drought stress treatment at the vegetative stage

Transgenic and NT rice plants were germinated on Murashige‐Skoog (MS) media in a growth chamber in the dark at 28°C for 3 days, followed by light conditions at 30°C for 2 days. Ninety seedlings of each transgenic line and NT control plants were transplanted to soil pots (4 × 4 × 6 cm; 3 plants per pot) and grown for 5 weeks in a glasshouse (16 h light/8 h dark) at 28–30°C. Drought stress was performed by withholding water from the pots for 3 days and irrigation was performed. Soil moisture was measured throughout the experiment to confirm similar water‐deficit conditions using a soil moisture sensor (SM150, Delta‐T Devices) for 3 days. Before drought treatment and after 2 and 3 days without watering, 10 pots per genotype were measured, respectively. For the JIP test (Redillas *et al*., 2011; Strasser *et al*., 2004), 6‐week‐old transgenic and NT plants were exposed to drought in large soil pots (6 kg of soil) for 12 days using a Handy‐PEA fluorimeter.

### Agronomic trait analysis of rice plants grown in a paddy field

To evaluate the agronomic traits of the transgenic and nontransgenic (NT) rice plants, NT plants and three independent T_3_ homozygous transgenic lines were planted in a paddy field at the Kyungpook National University, Gunwi (128:34E/36:15N), Korea. The experiment included three replicates where three different plots were planted in a randomized design. The yield components were of 30 plants per line from the three different plots for normal field conditions were then measured. To make the drought field conditions, plants were grown in a tank with a rain‐off shelter to cover rice plants from rain. To evaluate the yield parameters of the transgenic plants under drought field conditions, drought stress was imposed both before and after the heading stages, by removing the water. When the NT plants showed visual symptoms of drought stress (e.g. leaf rolling), they were irrigated and the same drought treatment repeated. After two drought stress treatments, the irrigated plants were grown until harvesting while maintaining irrigation. The yield components of 30 plants per each line from three different plots for the drought field conditions were measured.

### Subcellular localization of OsTF1L

For transient expression experiments, plasmids containing the *OsTF1L* coding sequence fused to the *GFP* reporter coding sequence were transformed into isolated rice protoplasts using PEG‐mediated transformation (Jung *et al*., 2015). For DAPI (Sigma, St. Louis, MO) staining, transformed rice protoplasts were incubated with 2 μg/mL DAPI for 2 min. GFP, chlorophyll and DAPI signals were detected using a Leica SP8 STED laser scanning confocal microscope (Leica, Solms, Germany).

### *In situ* hybridization

*In situ* hybridization experiments were performed as previously described (Lee *et al*., 2003), with minor modifications. Briefly, wild‐type rice plants (*Oryza sativa* L. var. Japonica cv. Illmi) were grown on MS media for 6 days, and shoots were fixed in FAA (50% ethanol, 5% acetic acid, 3.7% formaldehyde) for 20 min under vacuum at room temperature and for 12 h at 4°C without vacuum. Samples were dehydrated in a graded ethanol series, and finally embedded with paraplast (Sigma). The sections (8 μm) were made, and *OsTF1L* probes were prepared from the coding region and 3′ UTR (408 bp). Digoxigenin (DIG)‐labelled *OsTF1L* antisense and sense probes were generated by *in vitro* transcription with DIG‐labelled UTP (Roche, Mannheim, Germany) using the SP6 RNA polymerase and the T7 RNA polymerase, respectively (Roche).

### Phloroglucinol‐HCl staining

Hand‐cut cross sections of 3‐month‐old *OsTF1L* ^*OX*^, *OsTF1L* ^*RNAi*^ and NT stems were stained with phloroglucinol‐HCl as previously described (Jensen, 1962).

### Lignin extraction and quantification

Lignin of 1‐month‐old *OsTF1L* ^*OX*^, *OsTF1L* ^*RNAi*^ and NT leaves, roots and stems were extracted and quantified as previously described (Moreira‐Vilar *et al*., 2014). The lignin concentration was estimated using a standard curve generated with alkali lignin (Sigma‐Aldrich, St. Louis, MO).

### RNA‐seq

Total RNA was extracted from shoots of 2‐week‐old transgenic and NT plants using the RNeasy plant mini kit (Qiagen, Valencia, CA), according to the manufacturer's instruction. RNA quality and purity were assessed with a Thermo Scientific Nanodrop 2000 and an Agilent Bioanalyzer 2100. RNA‐seq libraries were prepared using the TruSeq RNA Library Prep Kit (Illumina, San Diego, CA) according to the manufacturer's instructions and sequenced (NICEM, Seoul National University, Korea) using the Illumina HiSeq2000 (Illumina). Single‐end sequences were generated and raw sequence reads were trimmed to remove adaptor sequences, and those with a quality lower than Q20 were removed using the clc quality trim software (CLCBIO, Aarhus, Denmark). All reads were assembled with the clc_ref_assemble 6 (version 4.06) program, using annotated gene and sequences from the rapdb (http://rapdb.dna.affrc.go.jp). Genes were defined as being differentially expressed if their transcript abundance was ≥2‐fold higher in *OsTF1L* ^*OX*^ compared to NT (Illmi).

### GO analysis

A total of 1743 genes that up‐regulated (>2‐fold) in the *OsTF1L* ^*OX*^ shoots as compared to the NT controls by RNA‐seq were subjected to AmiGO tool analysis to investigate gene ontology. GO analysis (*P* < 0.001) of the selected 1743 genes was performed using the PANTHER Classification System (http://amigo.geneontology.org/amigo/landing).

### ChIP‐seq and ChIP‐qPCR

ChIP analysis was performed as described in Bowler *et al*. (2004) using shoots of 2‐week‐old rice plants. To isolate protein/DNA complexes, shoots of *OsTF1L* ^*OX*^ and NT plants were rapidly cross‐linked in a buffer (0.4 m sucrose, 10 mm Tris‐HCl [pH 8.0], 5 mm β‐mercaptoethanol and 1% formaldehyde) in a vacuum desiccator for 15 min. The cross‐linked chromatin was isolated using a sucrose cushion, followed by random shearing for preparation of 100–300 bp of genomic DNA, using sonication. For immunoprecipitation, isolated and sheared chromatin was incubated with polyclonal anti‐myc (sc‐789; Santa Cruz Biotechnology, Dallas, Texas) and anti‐RNA Pol II (sc‐33754; Santa Cruz) antibody, while untreated samples were used as a control. Anti‐RNA Pol II antibody was used as a transcriptional control. The precipitated protein/DNA complex was collected by protein A agarose (Millipore 16‐266), and DNA was purified using the QIAquick PCR purification kit (Qiagen, Hilden, Germany). The ChIP‐Seq library was constructed using the TruSeq^®^ ChIP Sample Preparation Kit (Illumina), according to the manufacturer's instructions, and then sequenced using an Illumina Hiseq 2000 with 101 bp single‐end reads by NICEM (Seoul National University, Korea). Raw sequence reads were trimmed to remove adaptor sequences, and those with a quality lower than Q20 were removed using the clc quality trim software (CLCBIO). OsTF1L‐bound peaks were identified using peak scoring algorithm MACS (http://liulab.dfci.harvard.edu/MACS). We defined target genes as those that contain ChIP‐Seq peaks located within a 3‐kb region upstream from the transcription start site. For ChIP‐qPCR, the product of ChIP was analysed via quantitative PCR with a Mx3000P Real‐Time PCR system (Agilent Technologies, Santa Clara, CA). The relative enrichment was normalized with total input. All primer sequences are listed in Table S1.

### Transactivation assay

Full‐length *OsTF1L* and 3‐kb upstream sequences of target genes were amplified by PCR using high‐fidelity DNA polymerase PrimeStar (TaKaRa, Tokyo, Japan). *OsTF1L* was cloned into linearized *pHBT* vector containing a 35S promoter by *BamH*I and *Pst*I restriction enzyme using In‐Fusion HD cloning system (TaKaRa). Target genes promoter region were cloned into linearized *pGST6‐LUC‐NOS* vector harbouring a *GST6* enhancer/promoter‐FIREFLY LUCIFERASE by *BamH*I and *Nco*I restriction enzyme using In‐Fusion HD cloning system (TaKaRa). 15 μL of vector solution including 3 μg of effector, 1 μg of reporter and 0.5 μg of internal control was transformed into isolated rice protoplasts using PEG (polyethylene glycol)‐mediated transformation (Jung *et al*., 2015). The Dual‐Luciferase Reporter Assay System (Promega, Fitchburg, WI) was used to measure the luciferase activity according to manufacturer's manual, and the fluorescent values of luciferase were detected with an Infinite M200 System (Tecan, Seestrasse, Männedorf, Switzerland). Three independent transfections for each sample were performed, and the value of firefly luciferase was normalized to that of renilla luciferase. The *35S::rLUC* was used as an internal control.

### Scanning electron microscopy analysis

Leaves of 1‐month‐old NT and transgenic plants were detached. Stomatal density and aperture were measured using scanning electron microscopy (SEM) (Hitachi TM3030Plus, Europark Fichtenhain, Krefeld, Germany). For stomatal density analysis, average stomata numbers per square millimetre calculated from ten plants for each line. For comparison of the stomata length between NT and transgenic plants, twenty stomata were measured for each plant. Ten plants were used for each line. For stomatal aperture analysis, the two types (open and close) of stomata in the leaves of *OsTF1L* ^*OX*^, *OsTF1L* ^*RNAi*^ and NT plants were counted under normal and drought stress (30 min) conditions. Twenty stomata were measured for each plant. Ten plants were used for each line.

### Accession numbers

Genes from this article can be found in the National Center for Biotechnology Information (http://www.ncbi.nlm.nih.gov/) under the following accession numbers: GSE93287 (RNA‐seq), GSE93288 (ChIP‐seq), *poxN/PRX* (Os03g0235000), *PRX22* (Os01g0963000), *PRX2* (Os03g0434800), *CAD6* (Os04g0 229100) *CAD7* (Os04g0612700), *COMTL5* (Os04g0175600), *ABCG5* (Os03g0281900), *CYP450* (Os10g0513900). *ERF52* (Os05g0536250), *ERF101* (Os04g0398000), *HSP70* (Os03g027 7300), *LEA14* (Os01g0705200), *NHX4* (Os06g0318500), *PMEI* (Os12g0283400), *ABCG5* (Os03g0281900), *PME2* (Os01g0 311800), *PME6* (Os01g0788400), *ACA1* (Os03g0203700), *CAX2* (Os02g0138900), *GLR1.1* (Os09g0431100), *ONAC022* (Os03g0133000), *Nodulin protein* (Os12g0637800), *AAA‐type ATPase* (Os03g0802500), *DHHC4* (Os01g0925300) and *CASPL5B1* (Os01g0847300).

## Conflict of interest

The authors declare no conflict of interest.

## Supporting information


**Figure S1** Schematic drawing of the *OsTF1L* genomic structures.
**Figure S2** Vector maps for *OsTF1L* ^*OX*^, *OsTF1L* ^*RNAi*^ and *OsTF1L‐GFP*.
**Figure S3** ChIP‐seq and RNA‐seq analysis using shoots of *OsTF1L* ^*OX*^ plants.
**Figure S4** Soil moisture was measured throughout the experiment to confirm similar water‐deficit conditions for 3 days.
**Figure S5** Phenotypes of *OsTF1L* ^*OX*^ and *OsTF1L* ^*RNAi*^ transgenic rice plants under drought stress at the vegetative stage.
**Figure S6** qRT‐PCR analysis showing the transcript levels of lignin biosynthetic genes in 1‐month‐old *OsTF1L* ^*OX*^ and *OsTF1L* ^*RNAi*^ roots.
**Table S1** List of primers used in this study.
**Table S2** List of drought‐inducible genes up‐regulated in *OsTF1L* ^*OX*^ shoots compared with non‐transgenic shoots identified by RNA‐seq analysis.
**Table S3** List of stomatal movement related genes up‐regulated (>2 fold) in *OsTF1L* ^*OX*^ shoots compared with non‐transgenic shoots identified by RNA‐seq analysis.
**Table S4** List of lignin biosynthetic genes up‐regulated (>2 fold) in *OsTF1L* ^*OX*^ shoots compared with non‐transgenic shoots identified by RNA‐seq analysis.
