# Supplementary material for: Overexpression of OsTF1L, a rice HD‐Zip transcription factor, promotes lignin biosynthesis and stomatal closure that improves drought tolerance
Source: Plant Biotechnol J. 2018 Jul 19;17(1):118–31. doi: 10.1111/pbi.12951 (PMC6330637; doi:10.1111/pbi.12951)
Supplement: Supplementary file 1 — Figure S1 Schematic drawing of the OsTF1L genomic structures. Figure S2 Vector maps for OsTF1L OX , OsTF1L RNAi and OsTF1L‐GFP. Figure S3 ChIP‐seq and RNA‐seq analysis using shoots of OsTF1L OX plants. Figure S4 Soil moisture was measured throughout the experiment to confirm similar water‐deficit conditions for 3 days. Figure S5 Phenotypes of OsTF1L OX and OsTF1L RNAi transgenic rice plants under drought stress at the vegetative stage. Figure S6 qRT‐PCR analysis showing the transcript levels of lignin biosynthetic genes in 1‐month‐old OsTF1L OX and OsTF1L RNAi roots. Table S1 List of primers used in this study. Table S2 List of drought‐inducible genes up‐regulated in OsTF1L OX shoots compared with non‐transgenic shoots identified by RNA‐seq analysis. Table S3 List of stomatal movement related genes up‐regulated (>2 fold) in OsTF1L OX shoots compared with non‐transgenic shoots identified by RNA‐seq analysis. Table S4 List of lignin biosynthetic genes up‐regulated (>2 fold) in OsTF1L OX shoots compared with non‐transgenic shoots identified by RNA‐seq analysis. [file PBI-17-118-s001.docx]

**Supporting information**


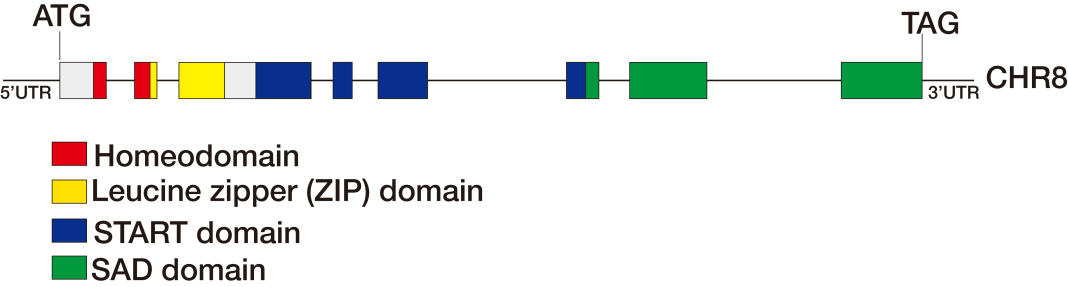


**Figure S1.** Schematic drawing of the OsTF1L genomic structures. The functional domains are colored as indicated. Filled box, exon; chromosome numbers (CHR) are shown.


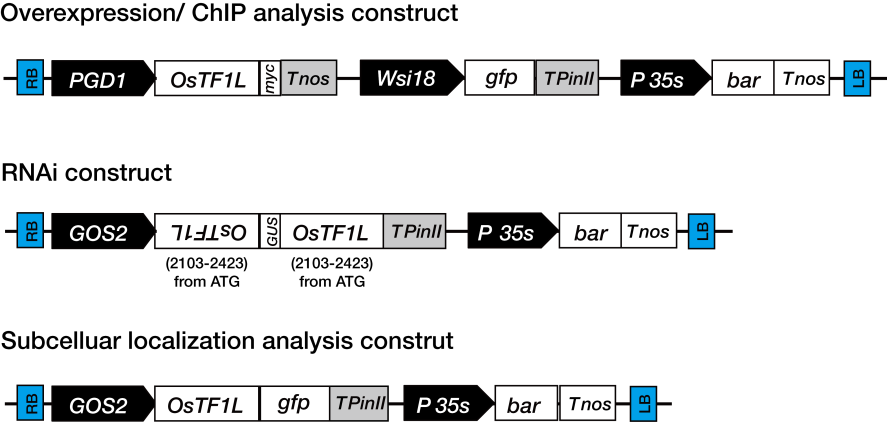


**Figure S2.** Vector maps for *OsTF1L^OX^*, *OsTF1L^RNAi^* and *OsTF1L-GFP*.


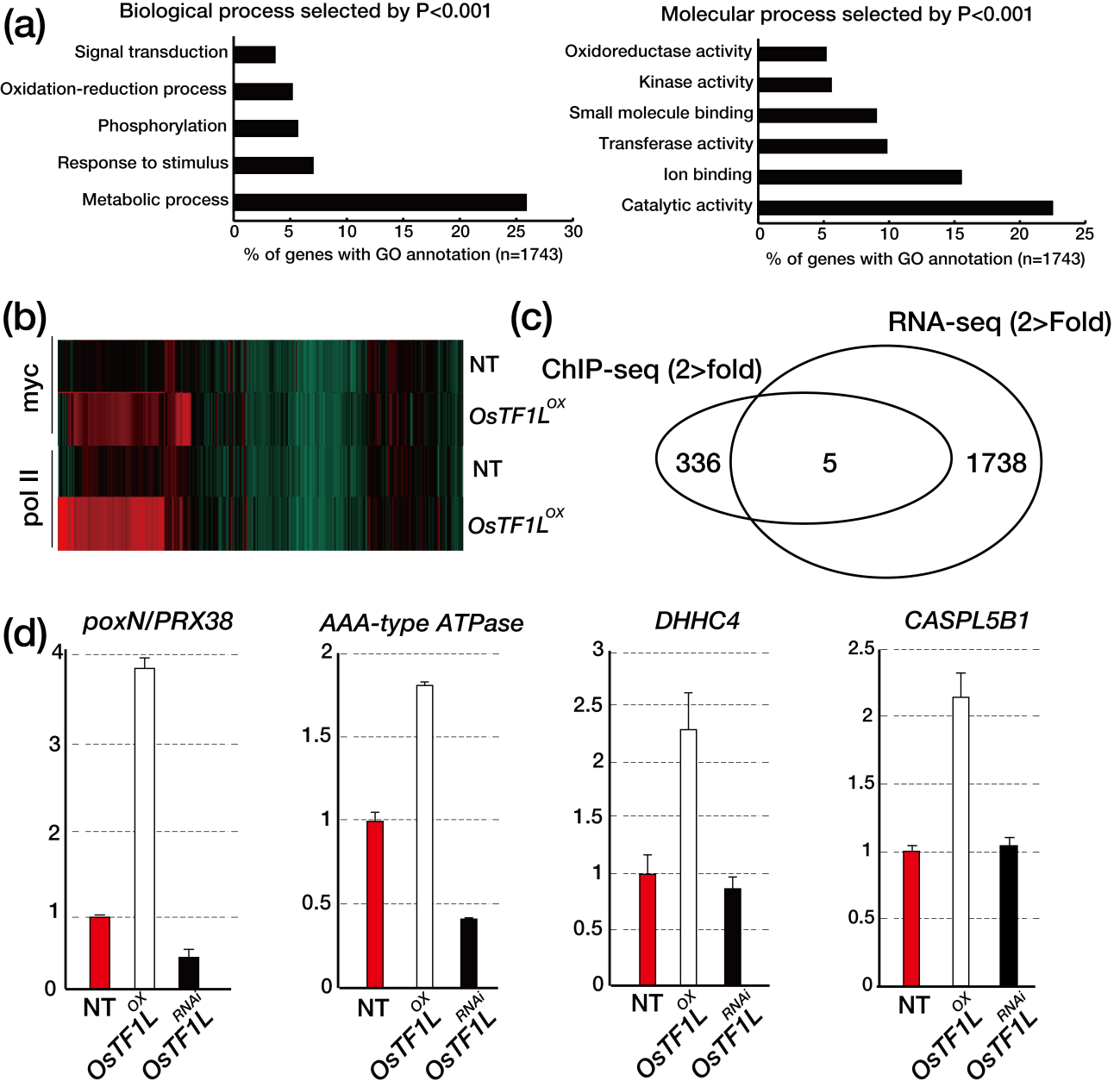


**Figure S3.** ChIP-seq and RNA-seq analysis using leaves of *OsTF1L^OX^* plants. **(A)** GO analysis (P < 0.001) of the selected 1743 genes using the PANTHER Classification System. **(B)** ).Heat map of all peak regions in the chromatin immunoprecipitation (ChIP)-seq data. **(C)** Venn diagram of the overlap between the ChIP-seq (using an anti-myc and anti-RNA Pol II antibody) and RNA-seq data (fold change > 2 **(D)** Transcript levels of the five direct targets of OsTF1L, which overlapped as being identified as targets in the chromatin immunoprecipitation (ChIP)-seq (with anti-myc and anti-RNA Pol II antibody) and RNA-seq analysis, in one-month-old *OsTF1L^OX^* and *OsTF1L^RNAi^* leaves. *Nodulin protein* was not detected due to basal expression levels very low.Ubi1 was used as the reference gene. Data are shown as the mean ± SD of three biological and two technical replicates.


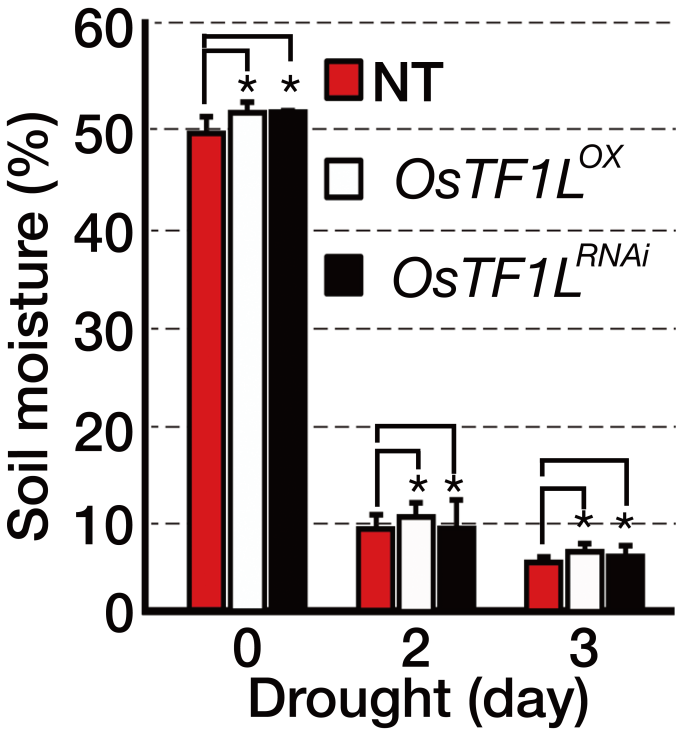


**Figure S4.** Soil moisture was measured throughout the experiment to confirm similar water-deficit conditions for 3 days. Data are means ± SD (n=10). Asterisks indicate significant differences compared with NT (*P* <0.05, Student’s *t*-test).


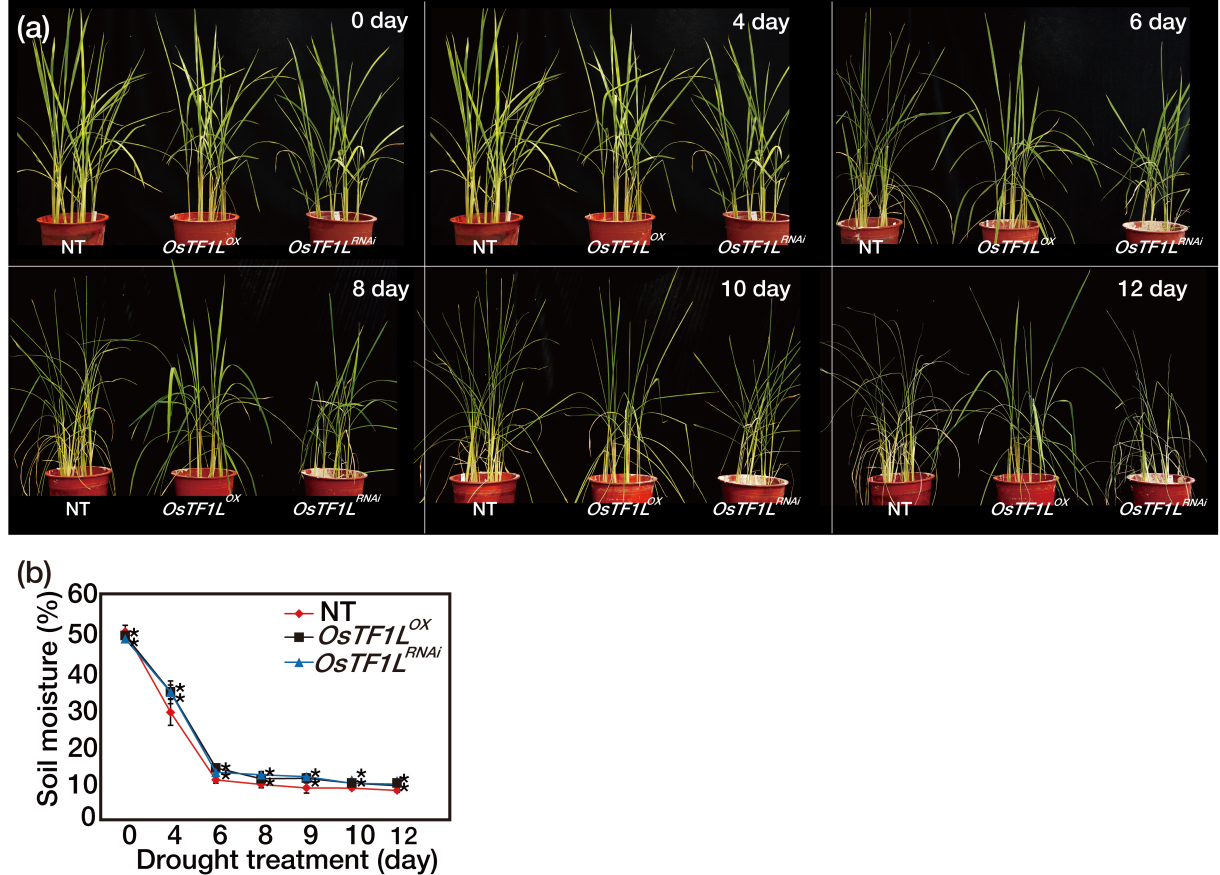


**Figure S5.** Phenotypes of *OsTF1L^OX^* and *OsTF1L^RNAi^* transgenic rice plants under drought stress at the vegetative stage. (A) Three independent homozygous T_3_ lines of *OsTF1L^OX^* and *OsTF1L^RNAi^* transgenic plants and non-transgenic (NT) control plants were grown in soil for 6 weeks and exposed to drought for 12 days. (B) Soil moisture was measured throughout the experiment to confirm similar water-deficit conditions. Data are means ± SD (n=10).

**
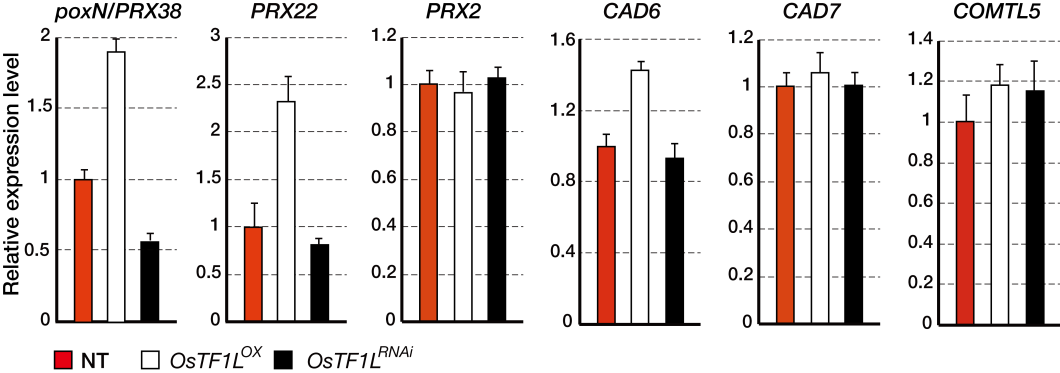
**

**Figure S6.** qRT-PCR analysis showing the transcript levels of lignin biosynthetic genes in one-month-old *OsTF1L^OX^* and *OsTF1L^RNAi^* roots. *Ubi1* was used as the reference gene. Data are shown as the mean ± SD of three biological and two technical replicates.

**Table S1.** List of primers used in this study.

| **Target Gene** | **Primer Sequence** | | |
| --- | --- | --- | --- |
|  | **Forward** | | **Reverse** |
| **qRT-PCR** | |  |  |
| *OsTF1L* (Os08g0292000) | | 5'-TGCGAACCTTCTAGTCCTAC-3' | 5'-GGTGAGGAGTTGGTATCTTC-3' |
| *poxN/PRX38* (Os03g0235000) | | 5'-TTGCTCGTCCTACACGAATC-3' | 5'-CAACGGAGTCGTTAGCATTC-3' |
| *PRX22* (Os01g0963000) | | 5'-GCGTCCAGGACTAGTGCTAC-3' | 5'-GGCAACTCGCGTCGTAGTAG-3' |
| *PRX2* (Os03g0434800) | | 5'-TGTCTGCCCCATTCATGGTC-3' | 5'-GAAGCAGTCGTGGAAGTGGA-3' |
| *CAD6* (Os04g0229100) | | 5'-ATGAAGGAGACGCAGGAGAT-3' | 5'-AGGCATGGAGGTTGGTACTA-3' |
| *CAD7* (Os04g0612700) | | 5'-CACATCCATGCACTCTTACC-3' | 5'-GGCGAAGTCGATCATCTCCT-3' |
| *COMTL5* (Os04g0175600) | | 5'-GATTGTCCTCCCGACGATCC-3' | 5'-CTCCAGCTCTGTCCTCTCCT-3' |
| *CYP450* (Os10g0513900) | | 5'-CACCACTGTGATGGCAAAGC-3' | 5'-CTGACATGTGAACCCCACCA-3' |
| *ERF52* (Os05g0536250) | | 5'-AGATGTTGTCATGTAGGCATCGG-3' | 5'-ACTTGAGAGCAGCAAGGTCA-3' |
| *ERF101* (Os04g0398000) | | 5'-TGCAATATGCATGGAGGGGT-3' | 5'-TCACACTTGAACAAAAGGAGGGA-3' |
| *HSP70* (Os03g0277300) | | 5'-TGATCCGATGTAGGTTGAGAGA-3' | 5'-ACGGAGTAATCATAGACACTGC-3' |
| *LEA14* (Os01g0705200) | | 5'-CCTACAAGCCTGGAACTGGG-3' | 5'-GCCCTCCATGGAACCAGAAA-3' |
| *NHX4* (Os06g0318500) | | 5'-CGCATGGCATAATGTGACGG-3' | 5'-ACGAATGCAGCTCTTCCCAA-3' |
| *PMEI* (Os12g0283400) | | 5'-CCAACCAGCTAACTACTCGCA-3' | 5'-GCCGCATGTACCTTTTGCAC-3' |
| *ABCG5* (Os03g0281900) | | 5'-CCTCTTCAGCGGCTTCTTCA-3' | 5'-GGCGTGTTGTCGAACATCTG-3' |
| *PME2* (Os01g0311800) | | 5'-CAAGGATTCGTGGCGGTGAA-3' | 5'-TGCAGTCCTGGAAAACCACG-3' |
| *PME6* (Os01g0788400) | | 5'-GTCAACTACAACGAGGGGCT-3' | 5'-TCTGATCTGCGCCATTAGCAT-3' |
| *ACA1* (Os03g0203700) | | 5'-ATTTCGCCAACACGACTCCT-3' | 5'-CTACCGACTTCAGTCCCGTG-3' |
| *CAX2* (Os02g0138900) | | 5'-GAAGCAAAAGCTTTGCCACG-3' | 5'-GAAAAGTGCCAGTTGCTCGG-3' |
| *GLR1.1* (Os09g0431100) | | 5'-AGCTTCTGGAGCAAAACCTGA-3' | 5'-ATTAATGACAGGGCGACAGGG-3' |
| *NAC022* (Os03g0133000) | | 5'-GGAAGAAGCAGACGTCACGG-3' | 5'-TAGAGCAGATGGGCGTAGGG-3' |
| *OsUBI1* (Os06g0681400) | | 5'-ATGGAGCTGCTGCTGTTCTA-3' | 5'-TTCTTCCATGCTGCTCTACC-3' |
| *AAA-type ATPase* (Os03g0802500) | | 5'-CGAGAAGGCCAAGGAGAATA-3' | 5'-CAATGGCGGCTAGCTACTTA-3' |
| *DHHC4* (Os01g0925300) | | 5'-TGGTTGGTTGTGAGGTATCC-3' | 5'-CTTGGAATGGCAGCTCAAGT-3' |
| *CASPL5B1* (Os01g0847300) | | 5'-GTGCTGTTCTCCTGAGCTTA-3' | 5'-GAGAGCAGATGTGGCACTAA-3' |
| **ChIP-qPCR** | |  |  |
| *poxN/PRX38*_P1 | | 5'-GAACCCAATTCTGCAACACCA-3' | 5'-ATTGACCTGGCCACCATCAC-3' |
| *poxN/PRX38*_P2 | | 5'-TCAATATCCGCTGCAAAAGGG-3' | 5'-AACCGCCTCGTCATGAACTT-3' |
| *poxN/PRX38*_P3 | | 5'-TAGCGTCCAATGACCCATCG-3' | 5'-TCGCATTGCTTCTTGCAGTG-3' |
| *Nodulin protein*_P1 | | 5'-AGCACGTGCATCAAGACCA-3' | 5'-CAAAATGCATGCTGCGAGGG-3' |
| *Nodulin protein*_P2 | | 5'-ACTTTCTATTCGGCTCATATGGT-3' | 5'-ACGCGCGTGAAAGTAGAGTA-3' |
| *Nodulin protein*_P3 | | 5'-TTCAGCGCCACAAAAGGTTC-3' | 5'-TACACGACGACAAGGGGAGT-3' |
| *AAA-type ATPase*_P1 | | 5'-GAAGGCATGATAGACCTCAC-3' | 5'-GCGCTGGACCTTAAGTAGTT-3' |
| *AAA-type ATPase*_P2 | | 5'-AGTAGGCAGCACCAGTTATG-3' | 5'-CTGATGCTGGAATCGCTTGG-3' |
| *AAA-type ATPase*_P3 | | 5'-GGAGGCCTTGTCGAAGAGGA-3' | 5'-CCGACTAGGCGGTGTCAGAA-3' |
| *DHHC4*_P1 | | 5'-TTGTCGCACGCACAAGAATC-3' | 5'-AGTAAGCAGATCGAGCGGTG-3' |
| *DHHC4*_P2 | | 5'-CACCGCTCGATCTGCTTACT-3' | 5'-GCTTGGCCGTGGAAGAAAGT-3' |
| *DHHC4*_P3 | | 5'-CCAGCACGTAGAGTAACGTA-3' | 5'-CAGTTGCGAGTCGTACATGG-3' |
| *CASPL5B1*_P1 | | 5'-CATCTGTTTTGTGCCGCTGA-3' | 5'-TGTTTTCGGATTGTGCAGGC-3' |
| *CASPL5B1*_P2 | | 5'-CACGAAATGCTTCGCCATCT-3' | 5'-TCCACTCGACACAACCTGC-3' |
| *CASPL5B1*_P3 | | 5'-ATTCTGGCGCAATCGATGTG-3' | 5'-GTCTACGCCGCTGTCAAGGT-3' |

**Table S2.** List of drought-inducible genes up-regulated in *OsTF1L^OX^* shoots compared with non-transgenic shoots identified by RNA-seq analysis.

| **Gene** | **ID** | **Log2 Ratio*** | | | **Log2 Ratio†** |
| --- | --- | --- | --- | --- | --- |
|  |  | **1d/C** | **2d/C** | **3d/C** |  |
| Conserved hypothetical protein | Os08g0240200 | 2.32 | 2.46 | 1.81 | 7.90 |
| Hypothetical gene | Os10g0142700 | 1.90 | 2.87 | 1.04 | 3.90 |
| OsWAK101 | Os10g0142600 | 1.87 | 2.91 | 1.02 | 3.47 |
| Gnk2 domain containing protein | Os01g0548750 | 1.00 | 5.48 | 7.91 | 3.24 |
| Hypothetical gene | Os05g0501001 | 1.00 | 2.32 | 2.17 | 3.12 |
| Conserved hypothetical protein | Os03g0772900 | 1.58 | 4.46 | 5.21 | 2.89 |
| OsLEA14 | Os01g0705200 | 1.42 | 7.84 | 8.12 | 2.88 |
| NB-ARC domain containing protein | Os07g0117900 | 1.74 | 3.42 | 3.81 | 2.45 |
| Conserved hypothetical protein | Os02g0209400 | 1.18 | 4.49 | 5.30 | 2.37 |
| OsPME2 | Os01g0311800 | 1.67 | 2.24 | 1.93 | 2.36 |
| Pectinesterase inhibitor | Os12g0283400 | 3.00 | 4.61 | 6.56 | 2.34 |
| Hypothetical protein | Os01g0616366 | 1.32 | 1.58 | 1.00 | 2.33 |
| Hypothetical gene | Os01g0800250 | 1.32 | 4.95 | 5.91 | 2.31 |
| HSP70 | Os03g0277300 | 1.44 | 4.70 | 7.13 | 2.31 |
| FH9 | Os08g0431200 | 2.00 | 5.83 | 8.60 | 2.28 |
| UDP-glucuronosyl/UDP-glucosyltransferase | Os05g0527000 | 1.07 | 4.26 | 5.39 | 2.12 |
| Conserved hypothetical protein | Os03g0724600 | 1.66 | 3.92 | 3.77 | 1.99 |
| reticuline oxidase | Os06g0549300 | 1.00 | 3.86 | 4.46 | 1.97 |
| Hypothetical protein | Os01g0723025 | 3.46 | 5.46 | 4.64 | 1.94 |
| DUF538 domain containing protein | Os07g0118200 | 3.70 | 6.95 | 6.48 | 1.85 |
| UDP-glucuronosyl/UDP-glucosyltransferase | Os05g0526900 | 2.58 | 6.08 | 6.09 | 1.79 |
| Oxidoreductase | Os07g0592100 | 1.04 | 4.28 | 4.83 | 1.69 |
| OsPR4b | Os11g0592100 | 1.00 | 1.55 | 1.68 | 1.68 |
| OsACE1, Mandelonitrile lyase | Os10g0524500 | 2.32 | 6.57 | 8.40 | 1.58 |
| Serine/threonine protein kinase | Os01g0621600 | 1.42 | 1.74 | 1.32 | 1.52 |
| Hypothetical protein | Os10g0111800 | 5.58 | 5.18 | 2.94 | 1.50 |
| DUF538 family protein | Os07g0120100 | 1.32 | 5.51 | 4.09 | 1.47 |
| NB-ARC domain containing protein | Os10g0136100 | 6.73 | 6.91 | 3.70 | 1.44 |
| OsAGP30 | Os02g0196100 | 1.00 | 4.25 | 3.00 | 1.38 |
| Hypothetical protein | Os05g0411150 | 1.00 | 3.00 | 4.56 | 1.37 |
| AP2/EREBP52 | Os05g0536250 | 3.39 | 3.81 | 6.29 | 1.35 |
| OsFbox546 | Os10g0326800 | 1.00 | 4.09 | 5.57 | 1.35 |
| Uncharacterised protein family UPF0497 | Os01g0847300 | 1.00 | 3.12 | 3.85 | 1.35 |
| Male sterility MS5 | Os09g0538500 | 1.00 | 2.58 | 2.32 | 1.35 |
| Embryo-specific protein | Os05g0569500 | 1.00 | 4.82 | 6.04 | 1.34 |
| OsHSP90 | Os04g0107900 | 1.62 | 3.44 | 2.44 | 1.34 |
| XA13 | Os08g0535200 | 1.42 | 4.68 | 2.66 | 1.34 |
| DUF3778 domain containing protein | Os01g0567200 | 1.14 | 1.00 | 3.10 | 1.33 |
| Hypothetical conserved gene | Os01g0727700 | 1.58 | 6.73 | 6.61 | 1.33 |
| OsNHX4 | Os06g0318500 | 1.58 | 6.36 | 6.09 | 1.31 |
| Histone H2B | Os09g0570850 | 1.66 | 2.91 | 3.60 | 1.30 |
| Chorismate mutase CM2 | Os08g0441600 | 1.85 | 1.00 | 2.81 | 1.29 |
| MAP65/ASE1 family protein | Os05g0552900 | 1.17 | 2.39 | 5.34 | 1.28 |
| Hypothetical gene | Os12g0125850 | 1.08 | 1.53 | 1.53 | 1.25 |
| Non-protein coding transcript | Os06g0600350 | 1.55 | 2.50 | 2.08 | 1.25 |
| FYVE/PHD-type domain containing protein | Os03g0187400 | 2.25 | 4.31 | 3.13 | 1.22 |
| Conserved hypothetical protein | Os11g0606001 | 2.81 | 3.17 | 3.00 | 1.22 |
| Oschib2 | Os10g0416800 | 2.54 | 3.98 | 8.22 | 1.19 |
| Aurora-B | Os01g0191800 | 1.00 | 2.00 | 2.00 | 1.18 |
| Sugar/inositol transporter | Os04g0511400 | 3.32 | 1.58 | 4.00 | 1.17 |
| OsTF1 | Os01g0788800 | 2.00 | 1.00 | 3.54 | 1.15 |
| agglutinin | Os01g0706800 | 1.06 | 3.00 | 1.79 | 1.13 |
| OsCOBL6 | Os07g0604400 | 1.70 | 3.04 | 3.93 | 1.09 |
| OsERF101 | Os04g0398000 | 1.00 | 4.89 | 4.65 | 1.09 |
| Late embryogenesis abundant protein | Os08g0163600 | 2.42 | 3.74 | 3.58 | 1.08 |
| RING/FYVE/PHD-type domain protein | Os03g0188200 | 1.17 | 3.21 | 4.07 | 1.06 |
| OsABCG5 | Os03g0281900 | 3.32 | 3.25 | 4.09 | 1.05 |
| B0403H10-OSIGBa0105A11.3 protein | Os04g0600100 | 1.04 | 1.17 | 2.57 | 1.02 |
| Cytochrome P450 family protein | Os10g0513900 | 1.06 | 1.45 | 1.89 | 1.01 |

*Log2 ratio between leaves of drought-treated/ not-treated controls in wild-type rice plant (Chung et al., 2016).

† Log2 ratio in *OsTF1L^OX^* leaves compared with non-transgenic leaves.

**Table S3.** List of stomatal movement related genes up-regulated (> 2 fold) in *OsTF1L^OX^* shoots compared with non-transgenic shoots identified by RNA-seq analysis.

| **Gene** | **ID** | **Fold change** |
| --- | --- | --- |
| Plant-specific cell wall organization |  |  |
| peroxidase19 | Os01g0787000 | 3.95 |
| peroxidase22 | Os01g0963000 | 2.13 |
| peroxidase99 | Os07g0156200 | 5.09 |
| peroxidase128 | Os10g0536700 | 3.88 |
| BRITTLE CULM1-like 6 | Os07g0604300 | 2.44 |
| BRITTLE CULM1-like 7 | Os07g0604400 | 2.12 |
| Expansin-A16 | Os06g0621900 | 3.76 |
|  |  |  |
| Cell wall modification |  |  |
| Pectin methylesterase 2 | Os01g0311800 | 5.14 |
| Pectin methylesterase 6 | Os01g0788400 | 2.36 |
| Pectin methylesterase 30 | Os11g0172100 | 2.62 |
| Pectinesterase inhibitor | Os04g0445300 | 2.00 |
| Pectinesterase inhibitor | Os06g0711800 | 3.93 |
| Pectinesterase inhibitor | Os12g0283400 | 5.07 |
|  |  |  |
| Nitrate transporter |  |  |
| Nitrate transporter 1.5 | Os02g0716800 | 3.04 |
| Nitrate transporter 2.4 | Os01g0547600 | 4.37 |
| Activator for nitrate transporter 2 | Os04g0480200 | 2.13 |
| Major facilitator superfamily 1 | Os03g0682100 | 2.75 |
|  |  |  |
| Ca2+ ATPase |  |  |
| Ca2+ P-Type ATPase 1 | Os03g0203700 | 3.98 |
| Ca2+ P-Type ATPase 9 | Os02g0176700 | 2.55 |
|  |  |  |
| Plasma H+ ATPase |  |  |
| Plasma membrane H+ P-Type ATPase 10 | Os06g0181500 | 2.56 |
|  |  |  |
| Glutamate receptor |  |  |
| Glutamate receptor like 1.1 | Os09g0431100 | 3.26 |
|  |  |  |
| Ca2+/H+ antiporter |  |  |
| Vacuolar cation/proton exchanger 2 | Os02g0138900 | 2.14 |
|  |  |  |
| ABCG family |  |  |
| ATP-binding cassette protein subfamily G member 5 | Os03g0281900 | 2.07 |
| ATP-binding cassette protein subfamily G member 35 | Os01g0609200 | 6.16 |
|  |  |  |
| Others |  |  |
| NAC domain-containing protein 022 | Os03g0133000 | 3.30 |

**Table S4.** List of lignin biosynthetic genes up-regulated (> 2 fold) in *OsTF1L^OX^* shoots compared with non-transgenic shoots identified by RNA-seq analysis.

| **Gene** | **ID** | **Fold change** |
| --- | --- | --- |
| C4H | Os01g0820000 | 2.53 |
| COMT | Os04g0175600 | 3.79 |
| CAD6 | Os04g0229100 | 11.66 |
| CAD7 | Os04g0612700 | 7.62 |
| UGT | Os07g0490100 | 13.50 |
| UGT | Os10s0442400 | 7.19 |
| UGT | Os01g0597800 | 4.97 |
| UGT | Os05g0527000 | 4.35 |
| UGT | Os01g0176000 | 4.29 |
| UGT | Os02g0207100 | 3.88 |
| UGT | Os05g0526900 | 3.45 |
| UGT | Os01g0805400 | 3.00 |
| UGT | Os09g0119600 | 2.59 |
| UGT | Os04g0556400 | 2.31 |
| UGT | Os04g0206500 | 2.15 |
| UGT | Os01g0734800 | 2.14 |
| UGT | Os03g0841600 | 2.09 |
| UGT | Os07g0241500 | 2.05 |
| UGT | Os08g0404000 | 2.03 |
| GH | Os09g0518000 | 3.04 |
| PRX | Os01g0787000 | 3.95 |
| PRX | Os01g0842500 | 3.94 |
| PRX | Os10g0536700 | 3.88 |
| PRX | Os05g0499300 | 3.81 |
| PRX | Os03g0235000 | 3.78 |
| PRX | Os03g0434800 | 2.59 |
| PRX | Os07g0676900 | 2.26 |
| PRX | Os01g0963000 | 2.13 |
| LACC | Os01g0842500 | 3.94 |

C4H, cinnamic acid 4-hydroxylase; COMT, caffeic acid O-methyltransferase; CAD, cinnamyl alcohol dehydrogenase; UGT, UDT-glycosyltransferase; GH, beta-glucosidase; PRX, peroxidase; Lacc, laccase.
